# Microfluidic Nanocomposite Lubricating Microgels Localized Deliver Celastrol for Remodeling the Immune Microenvironment to Enhance Osteoarthritis Treatment

**DOI:** 10.34133/research.1124

**Published:** 2026-02-27

**Authors:** Peng Guo, Qiaolin Yang, Wen Shi, Qin Yang, Yuchun Liu, Ya Tian, Wenbi Tuo, Xiaoli Yi, Jun Zhao, Siwei Xiong, Weidong Zhang, Rui Zeng, Chen Zhang, Yan Qu

**Affiliations:** ^1^ Chinese Medicine Germplasm Resources Innovation and Effective Uses Key Laboratory of Sichuan Province, Chengdu University of Traditional Chinese Medicine, Chengdu 611137, China.; ^2^ Key Laboratory of Research and Application of Ethnic Medicine Processing and Preparation on the Qinghai Tibet Plateau, Chengdu 610225, China.; ^3^ Sichuan Province Engineering Technology Research Center of Natural Small Molecule Drug, Tianfu TCM Innovation Harbour, Chengdu University of Traditional Chinese Medicine, Chengdu 611930, China.

## Abstract

In response to the disparity between the notable biological efficacy and systemic toxicity of celastrol (Cel), this study established a synergistic delivery system (Cel/Lipo+/BHMs) that integrates *Bletilla striata* polysaccharide (BSP) and hyaluronic acid biomimetic lubricating double-cross-linked microgels (BHMs) infused with Cel cationic liposomes (Cel/Lipo+). The objective is to collaboratively attain attenuation, sustained retention, and reconfiguration of the immunological milieu for the treatment of osteoarthritis. Cel/Lipo+ selectively targets chondrocytes via electrostatic interactions, hence improving Cel delivery and reducing off-target damage. Biolubrication by BHMs reduces friction and markedly extends the drug’s retention duration within the joint cavity. In vitro studies have shown that Cel/Lipo+/BHMs possess excellent biocompatibility, enhance the proliferation and migration of chondrocytes, diminish oxidative stress, and work in conjunction with Cel/BSP to modulate macrophage reprogramming while suppressing the release of proinflammatory factors tumor necrosis factor-α and interleukin-1β. In a rat osteoarthritis model induced by monosodium iodoacetate, Cel/Lipo+/BHMs effectively mitigated cartilage degeneration by synergistically suppressing the inflammatory response, diminishing the expression of the cartilage degradation enzyme matrix metalloproteinase 13, enhancing the synthesis of type II collagen and aggrecan, and ameliorating subchondral bone microstructure. The therapeutic efficacy markedly exceeds that of either Cel/Lipo+ or BHMs individually, thereby emphasizing the primary benefits of the multitiered collaborative mechanism of the delivery system.

## Introduction

Osteoarthritis (OA) is a chronic joint disorder resulting from the deterioration of cartilage [1,2]. The primary clinical signs are weight-bearing or stress-induced joint discomfort, stiffness, and restricted mobility, which may ultimately result in joint deformity and functional impairment, substantial affecting patients’ quality of life and imposing substantial social and economic burdens [3,4]. Existing OA therapy options exhibit significant limitations: Systemic medications, such as nonsteroidal anti-inflammatory drugs, glucocorticoids, and opioids, may reduce pain and inflammation; however, their long-term effectiveness is inadequate and linked to systemic side effects and addiction potential [5,6]. Oral cartilage-protecting medicines, including chondroitin and glucosamine, exhibit limited effectiveness and suboptimal long-term patient adherence [7]. Intra-articular injection of hyaluronic acid (HA) seeks to enhance lubrication; nevertheless, its viscosity significantly decreases at high shear rates, failing to mimic the boundary lubrication mechanism of natural phosphatidylcholine lipids, hence restricting its effectiveness [8,9].

The primary pathological features of OA are the progressive deterioration of articular cartilage and simultaneous synovial inflammation, which together act as the main drivers of disease progression [10,11]. Trauma or joint degeneration prompts chondrocytes to secrete damage-associated molecular patterns (DAMPs), including cartilage fragments [12]. DAMPs are recognized by pattern recognition receptors in synovial tissue, leading to the polarization of macrophages to the proinflammatory M1 phenotype and the activation of the classical nuclear factor κB (NF-κB) signaling cascade, resulting in the secretion of various proinflammatory proteins, including tumor necrosis factor-α (TNF-α), interleukin-1α (IL-1α), and IL-1β [13]. The inflammatory mediators, in conjunction with matrix metalloproteinases (MMPs) generated by activated M1 macrophages, directly influence chondrocytes, expediting the destruction of the cartilage matrix, particularly proteoglycans and type II collagen (Col2), resulting in cartilage injury [14]. The freshly formed cartilage fragments function as DAMPs, further enhancing macrophage activation and the inflammatory response, so creating a self-perpetuating cycle of oxidative stress, inflammation, and cartilage deterioration, which markedly accelerates the advancement of OA [15]. Moreover, this persistent inflammatory milieu generates excessive reactive oxygen species (ROS), leading to oxidative stress, which serves as a fundamental mechanism behind cartilage degradation [16,17]. ROS not only directly triggers chondrocyte apoptosis but also activates MMPs, further compromising matrix integrity and limiting chondrocyte synthesis of essential matrix components, such as proteoglycans and Col2, so directly undermining the structure and function of cartilage [18].

Celastrol (Cel) is a distinctive active compound found in the roots of the traditional Chinese medicinal plant *Tripterygium wilfordii* Hook F, which has a longstanding history of use in antirheumatic treatments [19,20]. Contemporary pharmacological research has validated that Cel demonstrates considerable anti-inflammatory effects in models of autoimmune arthritis [21,22]. In recent years, its efficacy in the management of OA has been elucidated [23]. Cel can efficiently inhibit the inflammatory cascade initiated by the key proinflammatory factor IL-1β, improve the inflammatory microenvironment of chondrocytes, reduce the expression of MMP-13, and promote the synthesis of Col2, thereby alleviating cartilage matrix degradation and aiding in repair [24]. Furthermore, its possible capacity to mitigate oxidative stress merits consideration. Nonetheless, the pronounced efficiency of Cel starkly contrasts with its considerable systemic toxicity, which is challenging to circumvent. Simultaneously, its intrinsic low water solubility and inadequate intestinal absorption result in exceedingly low oral bioavailability, thus restricting the expression of its therapeutic efficacy [25]. Therefore, it is imperative to address the constraints of its narrow therapeutic window and low bioavailability, as well as to develop safe and effective delivery systems for Cel to enhance its anti-inflammatory, antioxidant, and cartilage repair properties, which are urgent issues that necessitate prompt resolution. *Bletilla striata* polysaccharide (BSP) is a natural glucomannan derived from the traditional Chinese medicinal plant *B. striata* [26]. It possesses outstanding biocompatibility, degradability, and structural adaptability, making it an optimal matrix for the development of biomedical functional materials [27,28]. Polysaccharide hydrogel derived from BSP serves as a biomimetic material for the extracellular matrix (ECM), effectively replicating the milieu of the natural cartilage matrix and offering optimal 3-dimensional support for chondrocyte adhesion, proliferation, and matrix secretion [29,30]. Research indicates that BSP-based hydrogel scaffolds possess favorable physical qualities and can efficiently modulate macrophage activity while suppressing inflammatory-response-induced chondrocyte damage, therefore collaboratively enhancing chondrocyte repair and tissue regeneration [31,32]. Nonetheless, because of the intricate physiological and mechanical microenvironment within the articular cavity, it remains a significant challenge to create a BSP-based delivery system capable of achieving multidimensional synergistic regulation of joint function, including anti-inflammatory, proregenerative, lubricating, and sustained drug release effects.

The compact ECM of articular cartilage primarily consists of a robust collagen fiber network and proteoglycans abundant in highly sulfated glycosaminoglycans, creating a high-density physical structure and possessing significant negative charges [33,34]. This 2-fold barrier significantly restricts the infiltration of therapeutic agents and the efficacy of delivery to chondrocytes [35]. Cationic liposomes, possessing a positive surface charge, can efficiently surmount the negative electrical barrier of cartilage tissue via electrostatic attraction, therefore markedly augmenting drug penetration inside the matrix and facilitating targeted delivery to chondrocytes [35]. The injectable microgel technology effectively lubricates the joint cavity and minimizes joint wear due to its high moisture and 3-dimensional hydrophilic characteristics [36–38]. Furthermore, microgels serve as an intelligent sustained release platform, facilitating the continual release of preloaded cationic liposomes for the prolonged delivery of therapeutic agents [39–41]. Microgels can efficiently evade clearance by synovial fluid in joints [42–44]. Consequently, combining the effective penetration and targeting capabilities of cationic liposomes with the lubrication and sustained release properties of microgels to develop a synergistic delivery system present a promising approach to surmounting cartilage delivery challenges and enhancing the therapeutic efficacy for OA.

**Fig. 1.**
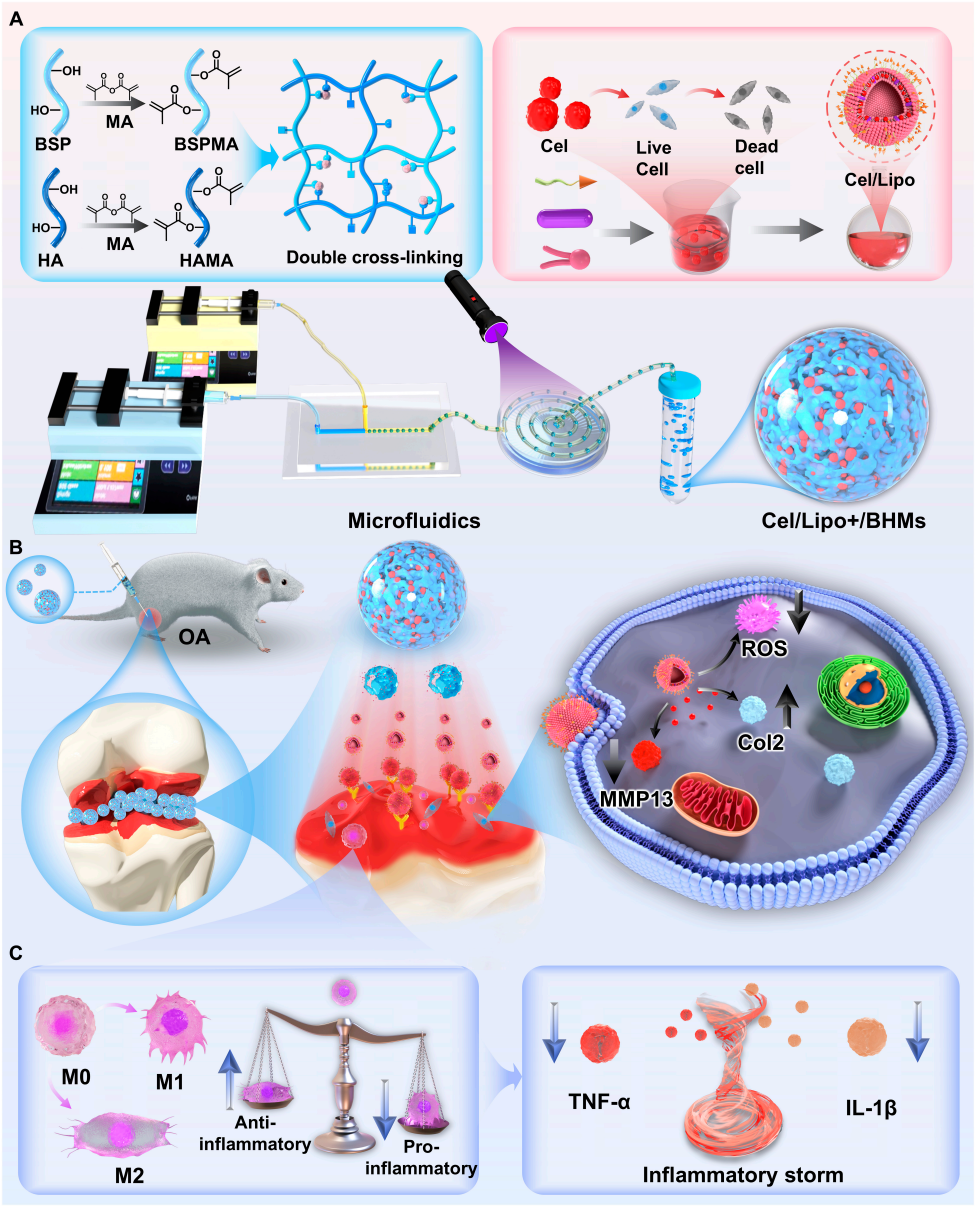
The preparation method of Cel/Lipo+/BHMs and the schematic representation of the OA model application. (A) BSP and HA were altered through a chemical modification strategy to create a double cross-linking system. Cationic liposomes were formulated using the thin-film dispersion method to mitigate toxicity and enhance the efficiency of Cel, while microfluidic technology was used to produce bearing lubrication microgels. Simultaneously, Cel/Lipo+ was incorporated to create Cel/Lipo+/BHMs. (B) Cel/Lipo+/BHMs were administered into the joint cavity. Throughout the rolling lubrication process, Cel/Lipo+ was persistently subjected to building a hydration layer, and the liberated Cel/Lipo+ infiltrated the negative barrier of the cartilage matrix via electrostatic contact to target chondrocytes and enhance their functionality. (C) Cel/Lipo+/BHMs facilitated the polarization of M0 macrophages to M2 through the delivery of Cel and BSP, resulting in macrophage reprogramming, diminished sustained release of inflammatory mediators during the inflammatory storm in the joint cavity, and enhanced chondrocyte function degradation mediated by inflammatory factors.

Because of the intricate pathological mechanisms of OA, this study has innovatively created a functionally integrated, stepwise delivery method for in situ articular cavity applications. The system’s foundation consists of a cationic liposome containing Cel, integrated within a robust, superlubricating microgel made from BSP and HA using a photocrosslinking method (Fig. 1A). Upon injection into the articular cavity, the BSP/HA double-cross-linked microgel emulates the composition of natural lubricant and the “bearing rolling” function, demonstrating exceptional bionic lubrication efficacy. Its superior mechanical qualities and minimal friction coefficient (COF) significantly diminish wear on the articular surface and safeguard the cartilage. The 3-dimensional network structure of the microgel functions as a protective reservoir, facilitating the controlled, prolonged release of cationic liposomes, so greatly diminishing potential systemic toxicity and augmenting local therapeutic efficacy. In the presence of the joint microenvironment, the released cationic liposomes can successfully surmount the substantial negative electric barrier of the cartilage matrix due to their surface positive charge, facilitating targeted penetration and sustained retention of the drug in deep tissue (Fig. 1B). Cel interrupts the detrimental cycle of inflammation-induced cartilage damage by diminishing oxidative stress in chondrocytes, suppressing the inflammatory response, and modulating essential signaling pathways; concurrently, it collaborates with the distinctive immune regulatory properties of BSP to reprogram macrophages and inhibit malignant inflammation (Fig. 1C). This cationic liposome–microgel dual-stage delivery method achieves the attenuation and synergistic impact of Cel through the incorporation of biomimetic lubrication, charge-mediated penetration, temporally regulated drug release, and drug-carrier synergy. It offers a novel way to address the challenges of drug administration in OA treatment, facilitating high-efficiency and low-toxicity synergistic therapy.

## Results and Discussion

### Preparation and characterization of Cel/liposome

**Fig. 2.**
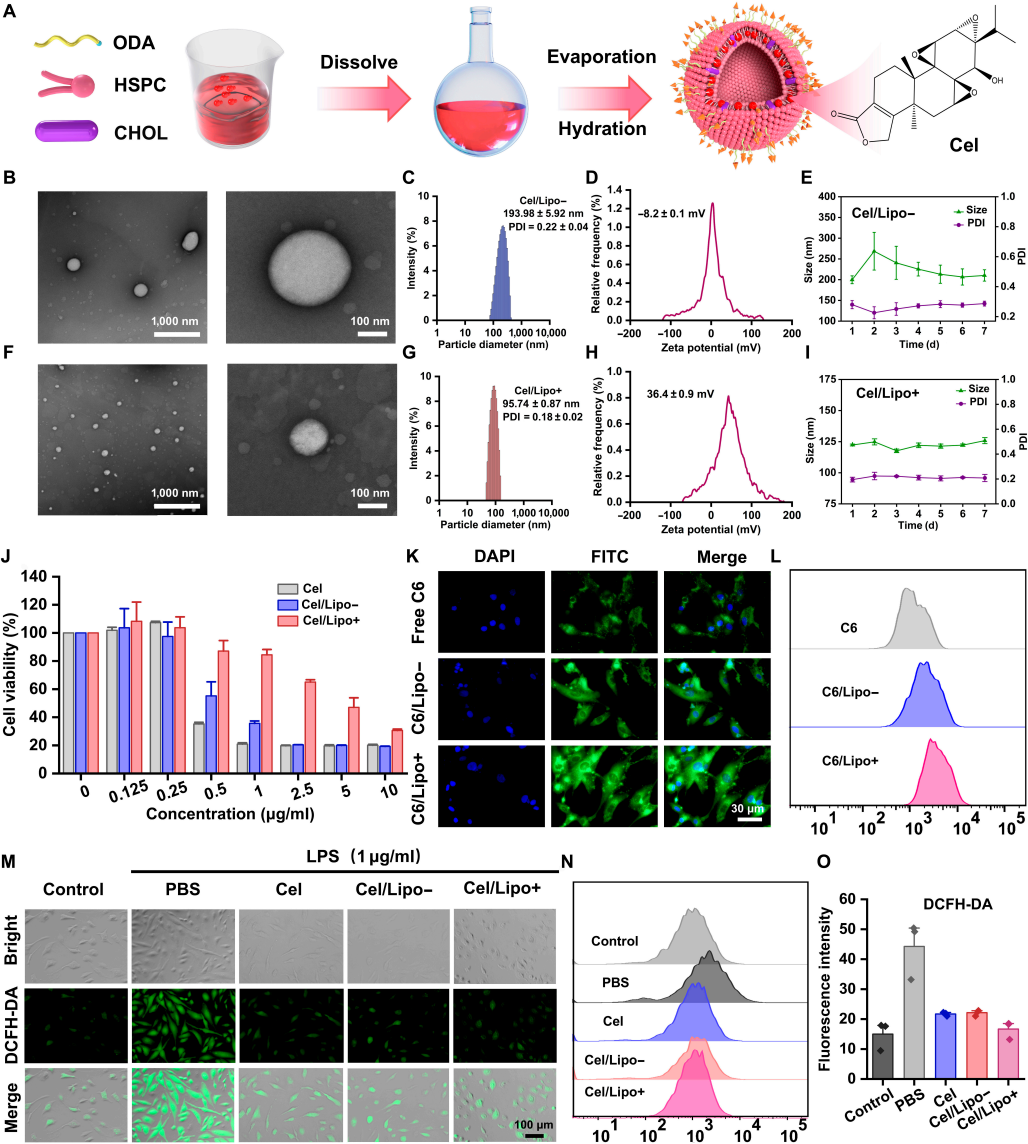
Preparation and characterization of Cel/Lipo+. (A) The schematic of Cel/Lipo preparation. ODA, octadecylamine; CHOL, cholesterol. (B) TEM images of anionic liposome Cel/Lipo−. Scale bars, 1,000 and 100 nm. (C) The particle size distribution of Cel/Lipo−. (D) Zeta potential of Cel/Lipo−. (E) Storage stability of Cel/Lipo−. (F) TEM images of cationic liposome Cel/Lipo+. Scale bars, 1,000 and 100 nm. (G) The particle size distribution of Cel/Lipo+. (H) Zeta potential of Cel/Lipo+. (I) Storage stability of Cel/Lipo+. (J) The cytotoxicity of different concentrations of Cel, Cel/Lipo−, and Cel/Lipo+ on C28/I2 cells was detected by the CCK-8 method. (K) Targeted enrichment of C28/I2 cells by free C6, C6/Lipo−, and C6/Lipo+. Scale bar, 30 μm. DAPI, 4′,6-diamidino-2-phenylindole. (L) Flow cytometry was used to detect the enrichment of C28/I2 cells by free C6, C6/Lipo−, and C6/Lipo+. (M) DCFH-DA was used to detect the removal of ROS in chondrocytes C28/I2 by free Cel, Cel/Lipo−, and Cel/Lipo+. (N) Flow cytometry was used to detect the scavenging effect of ROS in C28/I2 cells. (O) Quantitative statistics of DCFH-DA fluorescence detected by flow cytometry. Data are presented as means ± SD. *n* ≥ 3.

Cel exhibits substantial antioxidant properties. Research indicates that it increases the expression of antioxidant enzymes, efficiently eliminates ROS, and mitigates oxidative stress damage [45]. Nonetheless, Cel demonstrates dose-dependent toxicity. At elevated doses, it can lead to mitochondrial malfunction, trigger apoptosis, and result in organ damage, particularly affecting the liver and kidneys [46]. This study used lipid nanotechnology to encapsulate Cel in liposomes to mitigate its hazardous side effects (Fig. 2A). Hydrogenated soy phosphatidylcholine (HSPC) exhibits exceptional stability and is commonly utilized as a lipid substance, enhancing the stability of liposomes. Liposomes with varying charges encapsulating Cel were manufactured using the film dispersion method, and the ratios of the liposomal components were tuned to achieve appropriate surface charge and particle size, ensuring in vivo stability. Figure 2B illustrates that the transmission electron microscopy (TEM) micrographs of liposomes revealed that both types of liposomes displayed a spherical vesicle structure, with the cationic Cel/liposome (Cel/Lipo+) dispersion demonstrating greater uniformity (Fig. 2F). The particle size of the anionic Cel/liposome (Cel/Lipo−) was determined using dynamic light scattering. The size is 193.98 ± 5.92 nm, with a polydispersity index (PDI) of 0.22 ± 0.04 (Fig. 2C); the cationic Cel/Lipo+ particle size is 95.74 ± 0.87 nm, and its PDI is 0.18 ± 0.02 (Fig. 2G). The particle size and PDI were markedly smaller than those of Cel/Lipo− and aligned with the TEM data. The reduced particle size facilitated Cel/Lipo+ in overcoming the negative electrical barrier of cartilage. Figure 2D illustrates that the anionic Cel/Lipo− has a zeta potential of −8.2 ± 0.1 mV, while the Cel/Lipo+ exhibits a zeta potential of 36.4 ± 0.9 mV (Fig. 2H). The particle size and PDI results over 7 consecutive days indicate significant fluctuations in the anionic Cel/Lipo− particle size and PDI (Fig. 2E), whereas the cationic Cel/Lipo+ exhibits stability (Fig. 2I). This suggests that the incorporation of stearylamine not only enhances the positive potential of Cel/Lipo+ but also enhances the stability and dispersion of the nanosystem.

### Toxicity determination of Cel

The cytotoxicity of Cel, Cel/Lipo−, and Cel/Lipo+ was evaluated in vitro. Human normal chondrocytes C28/I2 were used as cellular models to ascertain if liposome encapsulation may markedly diminish the pronounced toxicity of Cel. Figure 2J illustrates that Cel, Cel/Lipo−, and Cel/Lipo+ exhibited pronounced dose-dependent toxic effects on C28/I2. The cell survival rate of Cel was merely 35.23% at a low dose of 0.5 μg/ml, indicating significant toxicity and adverse effects of Cel. At the same dose, the cell survival rates for the Cel/Lipo− and Cel/Lipo+ groups were 55.17% and 87.08%, respectively, both of which were considerably greater than that of the free drug Cel. The distinction between Cel/Lipo− and Cel/Lipo+ arises from the incorporation of stearylamine in Cel/Lipo+, which enhances the relative potential and stability of the nanostructure. Conversely, the Cel/Lipo− structure is prone to damage, leading to the release of Cel from the liposome and a significant increase in cell mortality. The toxicity test results indicated that the incorporation of the Cel/Lipo+ system significantly mitigated the toxic effects of Cel and augmented the therapeutic efficacy.

### Cel/Lipo+ enhanced chondrocyte targeting effect

The negative electrical barrier of cartilage has emerged as a significant impediment to medication delivery within the articular cavity [35]. The negative electrical barrier primarily consists of negatively charged glycosaminoglycans (e.g., chondroitin sulfate and HA) within the ECM, which inhibits conventional drugs from effectively permeating the cartilage layer and reaching chondrocytes due to homocharge repulsion [47,48]. To validate the targeted enrichment impact of Cel/Lipo− and Cel/Lipo+ on C28/I2 chondrocytes, we used the fluorescent detection probe coumarin-6 (C6) in lieu of Cel and utilized the identical manufacturing procedure to produce C6/Lipo− and C6/Lipo+. With the concentration and dosage of C6 maintained constant, they were cocultured with C28/I2 cells, and the enrichment was examined using a fluorescence microscope. Figure 2K illustrates that the fluorescence intensity of the C6/Lipo+ group was markedly greater than that of the C6/Lipo− and free C6 groups, suggesting that the cationic C6/Lipo+ utilized electrostatic interactions to specifically target chondrocytes. Furthermore, quantitative examination using flow cytometry indicated that C6/Lipo+ exhibited elevated fluorescence intensity, greatly surpassing that of the free C6 group (Fig. 2L). Consequently, it is demonstrated that the cationic Cel/Lipo+ can be concentrated on the cartilage surface through electrostatic interactions with the positive charges present, overcoming the charge barrier and infiltrating adjacent cartilage cells; its positive charge properties facilitate adhesion to negatively charged cartilage cell membranes and improve drug endocytosis efficacy.

### Cel/Lipo scavenging chondrocyte ROS analysis

To examine the impact of the liposome system on the antioxidative stress efficacy of Cel, we used lipopolysaccharide (LPS) to induce oxidative stress in C28/I2 chondrocytes and systematically assessed the ROS scavenging effects of Cel, Cel/Lipo−, and Cel/Lipo+ in C28/I2 cells. The intracellular ROS-sensitive fluorescent probe (2′,7′-dichlorodihydrofluorescein diacetate [DCFH-DA]) was utilized to assess the ROS levels in LPS-treated C28/I2 cells by fluorescence microscopy. The outcomes are illustrated in Fig. 2M. In comparison to the untreated group, the LPS-pretreated model group exhibited an elevated green fluorescence signal, signifying that LPS exposure provoked oxidative stress in C28/I2 cells. The fluorescence intensity of DCFH-DA in LPS-pretreated C28/I2 cells was markedly diminished following the introduction of Cel, Cel/Lipo−, and Cel/Lipo+. The fluorescent signal intensity of various groups was quantitatively assessed by flow cytometry (Fig. 2N). The findings exhibited a consistent pattern with those observed in fluorescence microscopy, as the fluorescence signals of Cel, Cel/Lipo−, and Cel/Lipo+ were markedly diminished (Fig. 2O). Consequently, liposome encapsulation did not influence the antioxidative stress efficacy of Cel, which further demonstrated that Cel/Lipo+ exhibited remarkable ROS scavenging properties in C28/I2 cells, effectively targeting excessive ROS in chondrocytes, mitigating oxidative-stress-induced cartilage degradation, and offering a novel approach for the precise management of degenerative joint disorders such as OA.

### Preparation and characterization of Cel/Lipo+/BSPMA-HAMA microgel precursor gel

Research indicates that BSP possesses remarkable biocompatibility and enzymatic degradation properties, while also effectively mimicking the topological structure of the ECM due to its abundant active groups, including hydroxyl and carboxyl moieties [49]. This endows it with a dual advantage as a multifunctional hydrogel carrier: It can create a biomimetic microenvironment through physical cross-linking and simultaneously enhance intrinsic pharmacological activities, such as anti-inflammatory effects and tissue repair. HA is a glycosaminoglycan consisting of d-glucuronic acid and *N*-acetylglucosamine. It is the primary constituent of connective tissue, including human joint synovial fluid. As a biological lubricant in bones and joints, it safeguards the ends of bones [50]. Intra-articular injection of HA is commonly used in the treatment of OA, effectively alleviating pain and enhancing synovial viscosity; however, the half-life of HA in the joint is brief, and repeated injections elevate the risk of adverse effects. Consequently, optimizing intra-articular injectable materials is crucial for facilitating OA treatment [51]. This study developed a BSP and HA double cross-linking system that not only emulates the ECM and modulates immune responses but also preserves the lubricating properties of HA in joints, thereby enhancing the intricate pathological microenvironment of OA.

To impart responsive molding capability to BSP and HA, we used an alkali-catalyzed esterification method for their chemical modification (Fig. S1). Under NaOH activation, the C6 hydroxyl group on the BSP molecular chain and the hydroxyl group on the HA molecular chain engage in a nucleophilic substitution reaction with methacrylic anhydride (MA), resulting in the successful incorporation of the photosensitive methacryloyl group to produce BSP and HA methacrylate derivatives, BSPMA and HAMA. This modification technique preserves the biological activity of BSP and HA while facilitating the formation of a photocrosslinked hydrogel system by ultraviolet (UV)-induced free radical polymerization.

BSPMA and HAMA were characterized by ^1^H nuclear magnetic resonance (Fig. S2A). The double peaks of 5.67/6.11 parts per million (ppm) in BSPMA and 5.71/6.14 ppm in HAMA confirmed the successful grafting of the methacryloyl group. The characteristic peaks of the polysaccharide main chain retain the integrity of the sugar ring. The substitution degrees of BSPMA and HAMA were 43% and 38%, respectively, calculated by the integral ratio of C1–H and double bond protons, which realized the controllable introduction of the methacryloyl group.

The chemical structures of BSP, BSPMA, HA, and HAMA were methodically examined using Fourier transform infrared spectroscopy (Fig. S2B). In the original spectrum of BSP, the extensive peak at 3,420 cm^−1^ corresponded to the stretching vibration of polysaccharide hydroxyl (–OH), the broad peak at 2,920/2,850 cm^−1^ represented the methylene vibration, the broad peak at 1,640 cm^−1^ indicated the bending vibration of adsorbed water δ (O–H), and the broad peak at 1,150/1,070 cm^−1^ was associated with the characteristic vibration of the glycosidic bond and pyranose ring. The distinctive *v* (C=O) vibration of the carboxylic acid group at 1,605 cm^−1^ and the δ (C–OH) vibration at 1,412 cm^−1^ define its structural identity. Post-MA modification, both BSPMA and HAMA exhibited a distinctive peak at 1,720 cm^−1^ for ester carbonyl ν (C=O), a C=C double bond vibration peak at 1,635 to 1,638 cm^−1^, and ester C–O–C antisymmetric and symmetric vibration peaks at 1,290/1,160 cm^−1^. The intensity of the carboxylic acid characteristic peak (1,605 cm^−1^) of HA markedly diminished postmodification, while the half-peak width of the hydroxyl vibration peak (3,420 cm^−1^) of BSPMA and HAMA decreased, respectively, indicating the involvement of the hydroxyl group in the esterification reaction. The intact preservation of the unique vibrational region of the polysaccharide framework signifies that the modification procedure does not compromise the primary structure of the sugar ring. The aforementioned synergistic characteristics collectively confirm the effective grafting of the MA group onto the 2 polysaccharides.

BSPMA and HAMA were effectively produced through chemical modification to impart photocuring characteristics. The small-bottle inversion experiment demonstrated that BSPMA, HAMA, and BSPMA-HAMA (B-HMA) could be effectively solidified to create hydrogel (Fig. S3A). The rheological and mechanical qualities of BSPMA, HAMA, and B-HMA pregels were examined by the assessment of their rheological characteristics. The storage modulus *G*′ and loss modulus *G*′′ of the pregel were evaluated experimentally. The magnitudes of the storage modulus *G*′ and loss modulus *G*′′ can indicate the condition and characteristics of the pregel. When *G*′>*G*′′, the system exhibits gel-like properties, signifying effective preparation of the pregel. The results indicate that the storage modulus *G*′ of the BSPMA pregel consistently exceeds the loss modulus *G*′′ (Fig. S3B), signifying the formation of a stable 3-dimensional cross-linked network within the BSPMA pregel, with the dynamic characteristics of the network exhibiting typical elastic-solid-dominated behavior. In the low-frequency domain, HAMA has solid-like characteristics, with the *G*′ surpassing the *G*′′ (Fig. S3C), signifying that the dynamic network established through physical entanglement and weak chemical cross-linking possesses elastic recovery capacity during gradual deformation. Currently, the molecular chain has sufficient time to release energy via the relaxation process, resulting in the material displaying gel characteristics. As the frequency escalates, *G*′′ surpasses *G*′, indicating the transformation mechanism of the augmented viscous dissipation of the material during rapid deformation. High-frequency short-term deformation causes the molecular chain to inadequately complete reconfiguration, leading to the unraveling of physical cross-linking points or the breakage of dynamic bonds. Simultaneously, the uncross-linked HA segment experiences viscous flow at elevated shear rates, resulting in energy dissipation as frictional heat. The *G*′ of BSPMA and HAMA double cross-linked gels consistently exceeded *G*′′ (Fig. S3D), while the loss modulus exhibited an upward trend relative to the single BSPMA system, suggesting that the dual network synergy facilitated a dynamic energy dissipation mechanism while preserving a highly elastic framework. The robust covalent cross-linking network of BSPMA offers rigid support to maintain the predominance of *G*′, whereas the weak dynamic cross-linking of HAMA experiences reversible dissociation and recombination during deformation, leading to local chain segment slippage or physical bond rupture, which further dissipates energy and elevates *G*′′.

### Preparation and characterization of Cel/Lipo+/BSPMA-HAMA microgel

**Fig. 3.**
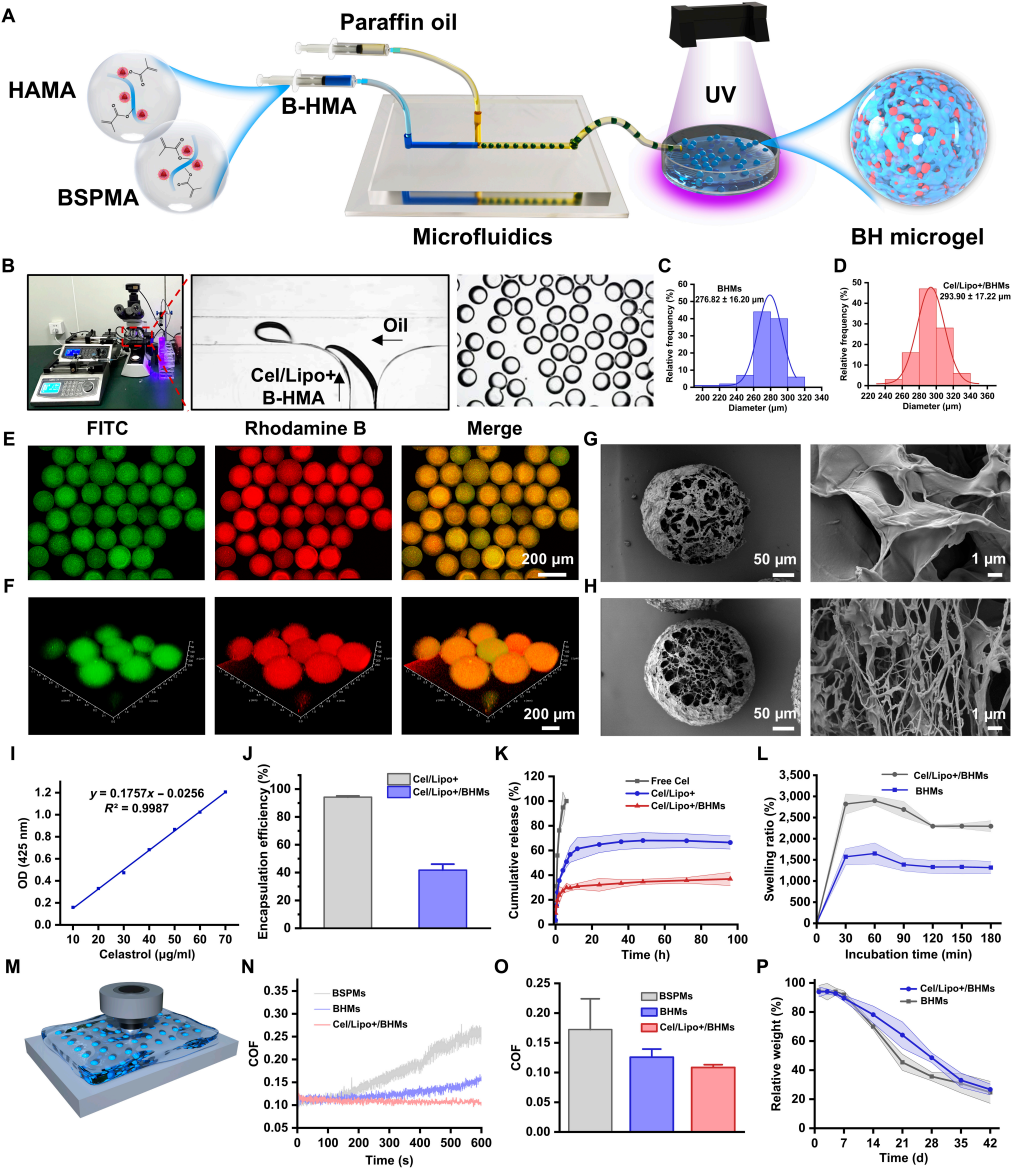
Preparation, characterization, and lubrication performance evaluation of Cel/Lipo+/BHM microgels. (A) BHM and Cel/Lipo+/BHM microgels were prepared by microfluidic technology. (B) Cel/Lipo+/BHM microgel generation, detection, and statistical system. (C and D) Particle size distribution of BHMs and Cel/Lipo+/BHMs. (E) Confocal laser scanning microscopy images of Cel/Lipo+/BHMs. Scale bar, 200 μm. (F) Cel/Lipo+/BHM laser confocal 3-dimensional reconstruction fluorescence images. (G and H) The SEM images of BHMs and Cel/Lipo+/BHMs were obtained. Scale bar, 50 and 1 μm. (I) Standard curves of Cel at different concentrations. OD, optical density. (J) Drug encapsulation efficiency of Cel/Lipo+ and Cel/Lipo+/BHMs. (K) Cel/Lipo+ and Cel/Lipo+/BHM in vitro drug release. (L) In vitro swelling curves of BHMs and Cel/Lipo+/BHMs. (M) Cel/Lipo+/BHM microgel friction and wear test diagram. (N) COF curves of BSPM, BHM, and Cel/Lipo+/BHM microgels. (O) Quantitative statistics of the COF of BSPM, BHM, and Cel/Lipo+/BHM microgels. (P) In vitro degradation curves of BHM and Cel/Lipo+/BHM microgels. Data are presented as means ± SD. *n* ≥ 3.

The BSPMA and HAMA double cross-linking method preserves the biological regulatory action of BSP while incorporating the joint lubricating efficacy of HA. Nevertheless, the direct administration of the gel system via intra-articular injection will augment the joint load, and the injected gel will not provide enhanced lubricating efficacy. Microgel is a 3-dimensional spherical hydrogel network, measuring in micrometers, characterized by an extensive specific surface area and a high degree of ECM mimicry [52]. Drawing from the rolling lubrication of bearings, intra-articular injection of microgels can provide rolling lubrication, diminish joint friction and wear, and prevent the functional deterioration of articular cartilage. The cationic Cel/Lipo+ composite BSPMA and HAMA double cross-linked microgel system (Cel/Lipo+/BHM) was synthesized using microfluidic technology (Fig. 3A). The technology is compatible with Cel/Lipo+ electrostatic targeting, lipid hydration lubrication, and microgel rolling lubrication, which can effectively ameliorate the pathological issues associated with joint wear. Prior research has validated that BSPMA and HAMA exhibit photoresponsive gel characteristics. Consequently, as illustrated in Fig. 3B, the aqueous solution including Cel/Lipo+, B-HMA, and lithium phenyl-2,4,6-trimethylbenzoylphosphinate serves as the dispersed phase, while 5% Span80 functions as the continuous phase. The precursor solution generates a monodisperse droplet within the polydimethylsiloxane chip due to the laminar shear effect, subsequently curing the cross-linking of the experimental droplets through UV-induced free radical polymerization. Optimal control of microgel shape is attained by refining the 2-phase hydrodynamic parameters. Optical microscopy and particle size analysis demonstrated that the microgels produced via microfluidic technology had a consistent spherical morphology and a restricted particle size distribution. The particle sizes of BHMs and Cel/Lipo+/BHMs were 276.82 ± 16.20 and 293.90 ± 17.22 μm, respectively (Fig. 3C and D). The disparity arises because the incorporation of Cel/Lipo+ elevates the viscosity of the precursor solution, hence augmenting the particle size of Cel/Lipo+/BHMs. To ascertain the spatial distribution characteristics of Cel/Lipo+ within microgels, we specifically stained the Cel/Lipo+ liposome membrane (fluorescein isothiocyanate [FITC], green) and the microgel network (rhodamine, red) using a dual fluorescence labeling technique, followed by colocalization analysis via laser confocal imaging. Figure 3E illustrates a consistent green fluorescence signal within the microgel, exhibiting a distinct spatial overlap with the red fluorescence, thus validating the successful encapsulation of Cel/Lipo+ within the 3-dimensional network. To further examine the spatial distribution properties of the carrier, *z*-axis layer scanning 3-dimensional reconstruction (Fig. 3F) was conducted on an individual microgel. The results indicated that the fluorescence signal of Cel/Lipo+ was isotropic in 3-dimensional space, with no discernible surface enrichment or segregation, suggesting that the Cel/Lipo+ and BHM microgel network established a robust interpenetrating structure. Scanning electron microscopy (SEM) micromorphology analysis revealed that BHM and Cel/Lipo+/BHM microgels had a highly linked 3-dimensional porous network structure both on the surface and internally (Fig. 3G and H). This interpenetrating network, characterized by a large specific surface area, can markedly enhance the loading efficiency of active ingredients and establish a 3-dimensional drug diffusion pathway that facilitates the controlled release of Cel. The distribution of Cel/Lipo+ is concurrently observable within the Cel/Lipo+/BHM microgel network.

### Cel/Lipo+/BHM microgel drug encapsulation and controlled release

Cel, being a category of poorly soluble triterpenoids, encounters numerous obstacles in therapeutic application. The bioavailability is limited, the therapeutic window is narrow, the toxicity and side effects are significant, and it is rapidly eliminated from the body due to poor water solubility [46]. This work used cationic liposome encapsulation technology to develop a Cel/Lipo+ delivery method to enhance the situation. This method markedly enhanced the water solubility of the medication while simultaneously facilitating its sustained release via the prolonged release properties of the phospholipid bilayer. In addition, Cel/Lipo+ was encapsulated within BHM microgels to create a Cel/Lipo+/BHM composite delivery system. The 3-dimensional network structure significantly improves the physical stability of the delivery system and mitigates the risk of rapid drug release, thereby ensuring long-term treatment for OA oxidative stress and chronic inflammation. The experimental data (Fig. 3I and J) indicated that the encapsulation efficiency of Cel/Lipo+ was 87.38 ± 10.51%, but the encapsulation efficiency of Cel/Lipo+/BHMs diminished significantly to 76.99 ± 10.01% following the second encapsulation of BHMs. This discrepancy may be attributed to 2 technical factors. Initially, during the elimination phase of organic solvents in microsphere synthesis, certain liposomal structures may disintegrate, resulting in drug leakage. Second, the mechanical stress during the cross-linking process of microspheres may induce localized damage to the liposome membrane structure. It is noteworthy that despite a reduction in encapsulation efficiency, the in vitro release experiment (Fig. 3K) demonstrates that Cel/Lipo+/BHMs possess superior sustained release characteristics; its release curve displays typical biphasic kinetic behavior: The rapid release phase within the initial 24 h offers immediate therapeutic concentrations for OA, while the subsequent sustained release phase maintains effective blood concentrations. In comparison to free Cel and Cel/Lipo+, the cumulative release of Cel/Lipo+/BHMs was merely 34.84%, thereby demonstrating that the incorporation of BHMs can significantly modulate the drug release kinetics. The multistage delivery approach of Cel/Lipo+/BHMs exhibits considerable advantages in the context of OA treatment. The micron-sized particle dimension can extend the retention duration of the formulation within the articular cavity. The system can circumvent the swift clearance of conventional liposomes caused by enzymatic hydrolysis or osmotic pressure fluctuations in the intricate OA environment through the “liposome–microgel” dual-controlled release mechanism, thereby overcoming the technical limitation of inadequate sustained release duration of single-layer liposomes.

### Biolubrication effect of Cel/Lipo+/BHMs

Inadequate lubrication heightens friction on the joint surface, worsening cartilage deterioration and the progression of OA [53,54]. Porous Cel/Lipo+/BHMs not only efficiently transport Cel/Lipo+ but also augment the lubricating efficacy to diminish joint wear. To assess the lubricating efficacy of Cel/Lipo+/BHM microgels, we used a high-frequency reciprocating friction and wear tester to conduct a friction test for 600 s (Fig. 3M). The COF of BSPMA microgels was 0.17, whereas the COF of BSPMA and HAMA double-cross-linked BHM microgels decreased to 0.12. The enhancement in lubricating efficacy is attributable to the hydrophilic group inside the HA molecular chain. The superior water retention capability prompts water molecules to establish a stable hydration layer. Throughout the friction process, despite the microgel’s wear, the hydration layer will remain exposed on the microgel’s outer surface to diminish the COF (Fig. 3N and O). Furthermore, the incorporation of Cel/Lipo+ diminishes the COF of Cel/Lipo+/BHM microgels to 0.10, attributable to the stable lipid bilayer formed by HSPC in Cel/Lipo+. Simultaneously, the hydrophilic terminus of the phospholipid molecule establishes a lipid hydration layer with adjacent water molecules, thus augmenting the system’s lubricating efficacy. Friction and wear testing indicate that Cel/Lipo+/BHMs can diminish friction and wear while significantly enhancing joint lubrication.

### In vitro swelling and degradation properties of Cel/Lipo+/BHMs

The microgel, being a 3-dimensional spherical network of polymeric entities, rapidly absorbs water, swells in solution, and retains substantial quantities of water [55]. In vitro swelling experiments demonstrated that both BHM and Cel/Lipo+/BHM microgels had superhydrophilic characteristics in deionized water (Fig. 3L). They could rapidly assimilate a substantial volume of water upon contact and exhibited a significant swelling rate. The swelling ratio of BHMs attained 1,573.20% in 30 min, but the swelling ratio of Cel/Lipo+/BHMs was 2,819.32%. The rapid liquid absorption characteristic arises from the abundant hydroxyl and carboxylic acid groups in the BSPMA and HAMA molecular chains, which capture water molecules via hydrogen bonding. In addition, the capillary effect produced by the 3-dimensional porous network structure enhances liquid penetration. The elevated swelling ratio of microgels has significant benefits in bioengineering applications, enabling complete drug absorption within the 3-dimensional network structure, hence enhancing the adsorption and prolonged efficacy of these active compounds.

The biodegradability of biological scaffold materials is a critical criterion for assessing biological materials, directly influencing the application of these scaffolds [56]. Biomaterials with varying biodegradation rates must be engineered for the healing of distinct tissues [57]. The biodegradability of microgels is crucial as a 3-dimensional network structure for intra-articular injection [39]. Excessive degradation will result in less deposition of ECM in articular cartilage tissue, and the swift disintegration of microgels causes rapid leakage of liposomes in Cel/Lipo+/BHMs, hence lowering the bioavailability of medication components and exacerbating side effects. Simultaneously, excessively slow breakdown will impede the creation and dispersion of cartilage. To assess the synergistic effect between the degradation behavior of microgels and the controlled release of pharmaceuticals, we investigated the degradation kinetics of microgels by recreating the microenvironment of the osteoarthritic articular cavity in vitro. Figure 3P illustrates that the degradation rates of BHMs and Cel/Lipo+/BHMs after 14 d were 70.00% and 78.18%, respectively, consistent with the degradation kinetics model. They had significant degradation within the initial 14 d and were entirely damaged by 42 d. The degradation rate was demonstrated to align with the rate of cartilage production, which holds substantial importance for OA treatment and the enhancement of cartilage regeneration.

### Biocompatibility evaluation of Cel/Lipo+/BHMs

**Fig. 4.**
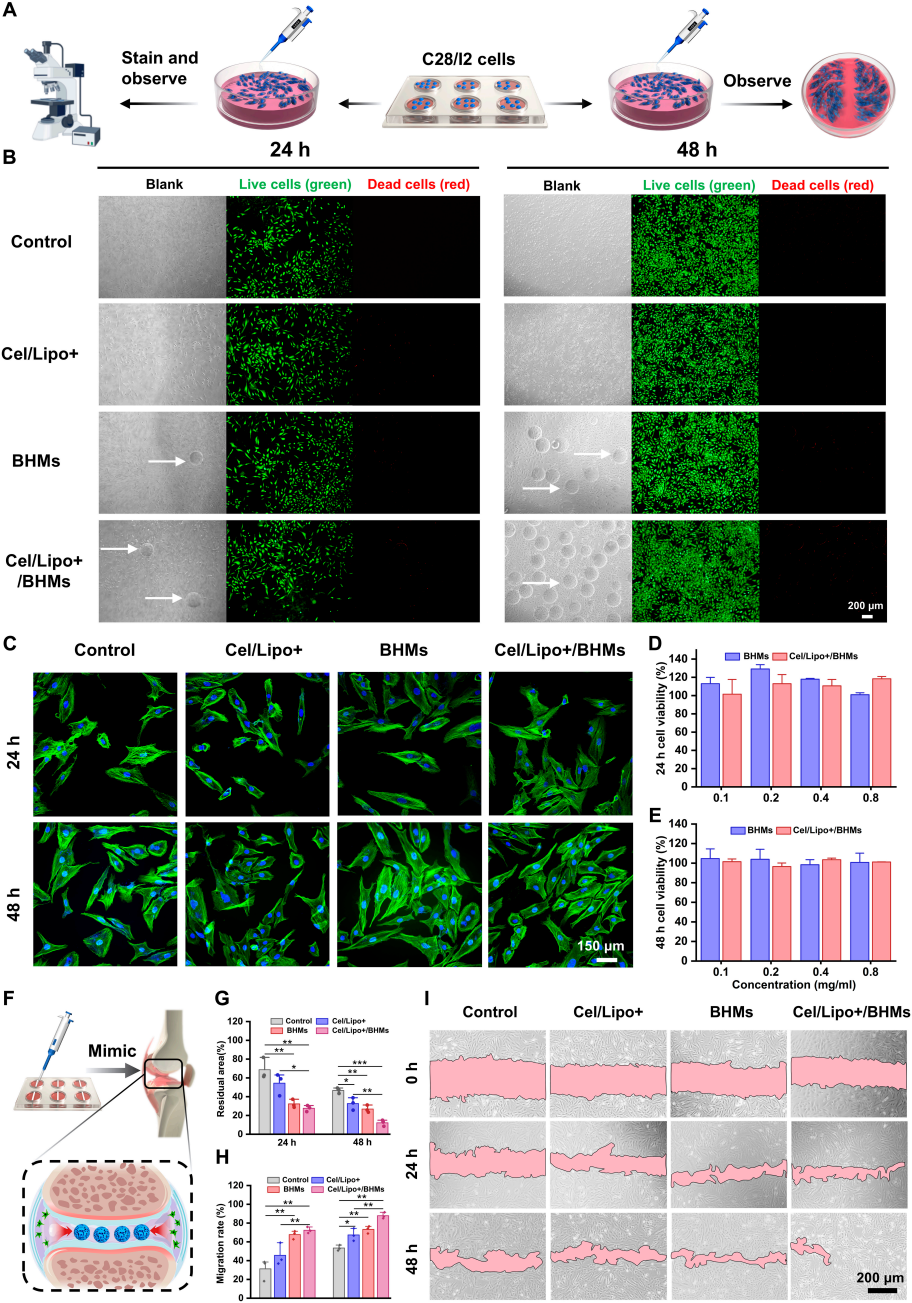
The biocompatibility and chondrocyte proliferation and migration activity of Cel/Lipo+/BHMs. (A) Cell experiment diagram. (B) Live/Dead staining of C28/I2 chondrocytes cocultured with different samples (control, Cel/Lipo+, BHMs, and Cel/Lipo+/BHMs). Scale bar, 200 μm. (C) C28/I2 cytoskeleton staining. Scale bar, 150 μm. (D and E) The CCK-8 method was used to detect the cell viability of chondrocytes cocultured with different concentrations of BHMs and Cel/Lipo+/BHMs for 24 and 48 h. (F) Schematic diagram of promoting articular cartilage cell migration. (G and H) Quantitative statistics of the residual area and migration rate of C28/I2 cells after treatment with different samples. (I) Cell migration results of C28/I2 cells treated with different samples. Scale bar, 200 μm. Data are presented as means ± SD. *n* ≥ 3. *P < 0.05, **P < 0.01, and ***P < 0.001.

Given that Cel/Lipo+/BHM microgels will directly interact with articular chondrocytes as an implant material for joint injection, it is imperative to assess the biosafety of these microgels. Prior research indicated that Cel exhibited considerable cytotoxicity at a dose of 0.5 μg/ml, whereas Cel/Lipo+ significantly mitigated the toxicity of Cel upon encapsulation. Consequently, the biocompatibility of Cel/Lipo+/BHM microgels will undergo further assessment by in vitro tests. In vitro cellular investigations (Fig. 4A) involved coculturing Cel/Lipo+, BHMs, and Cel/Lipo+/BHMs with C28/I2 human normal chondrocytes, whereas the control group received an equivalent volume of phosphate-buffered saline (PBS) for durations of 24 and 48 h. Live/Dead cell staining (Fig. 4B) demonstrated that all treatment groups exhibited a substantial quantity of viable cells (green) and negligible cell death (red). Cells in direct contact with BHM and Cel/Lipo+/BHM microgels exhibited no damage under bright-field microscopy. Simultaneously, 48-h green fluorescence verified that all groups exhibited identical cell morphology. Cell Counting Kit-8 (CCK-8) studies demonstrated the cell survival rates of BHMs and Cel/Lipo+/BHMs at various concentrations above 80% (Fig. 4D and E), indicating an absence of cytotoxic effects. Furthermore, in light of the potential for systemic exposure following joint injection, we assessed blood compatibility with a hemolysis test. The findings (Fig. S4A and B) indicated that Cel/Lipo+/BHMs exhibited a minimal hemolysis rate. The aforementioned results demonstrate that the microgel can significantly diminish the intrinsic toxicity of Cel and offer superior biocompatibility for cartilage-targeting applications.

### In vitro study of Cel/Lipo+/BHMs promoting chondrocyte proliferation and migration

Chondrocyte growth and migration are crucial in OA. Chondrocytes, the sole functional cells in articular cartilage, preserve the structure and function of cartilage through the synthesis of ECM [58]. During the advancement of OA, the deterioration of the ECM (including the degradation of Col2 and the loss of proteoglycans) and the inflammatory milieu will elicit a compensatory response from chondrocytes, characterized by attempts at repair through proliferation and migration. Chondrocyte proliferation may offset cell loss in the injured region by increasing cell numbers and forming clusters in the initial phase to secrete a provisional ECM. However, these proliferating cells experience phenotypic alterations due to chronic inflammation and oxidative stress in the microenvironment, resulting in aberrant matrix synthesis or fibrosis. Migration prompts chondrocytes to relocate to the injured region to repair the defect; however, the avascular nature of articular cartilage, its dense ECM structure, and heightened inflammatory factor activity significantly impede cellular mobility. Consequently, augmenting the proliferation and directional migration of chondrocytes will facilitate the expeditious treatment of OA. Figure 4C illustrates that phalloidin cytoskeletal labeling revealed the shape and proliferation status of C28/I2 chondrocytes following coincubation with Cel/Lipo+/BHMs, indicating an increased cell density within the same visual field, hence confirming the normal proliferation of C28/I2 cells. The BSP and HA in the microgel supply matrix nutrients, resulting in a higher density trend in cell proliferation compared to the control group. This demonstrated that Cel/Lipo+/BHMs enhanced chondrocyte proliferation. Furthermore, as illustrated in Fig. 4F, cell scratch assays were conducted to replicate the migration of chondrocytes within the articular cavity matrix. Figure 4I demonstrated that the migratory area in the BHM and Cel/Lipo+/BHM groups was markedly bigger than in the other groups. Simultaneously, in quantitative data (Fig. 4G and H), Cel/Lipo+/BHMs exhibited the minimal residual area of wound healing (12.14%) and the maximal wound migration rate (87.86%), signifying that Cel/Lipo+/BHMs augmented the migratory capacity of chondrocytes.

### Regulating oxidative stress and macrophage polarization to reshape the inflammatory immune microenvironment

**Fig. 5.**
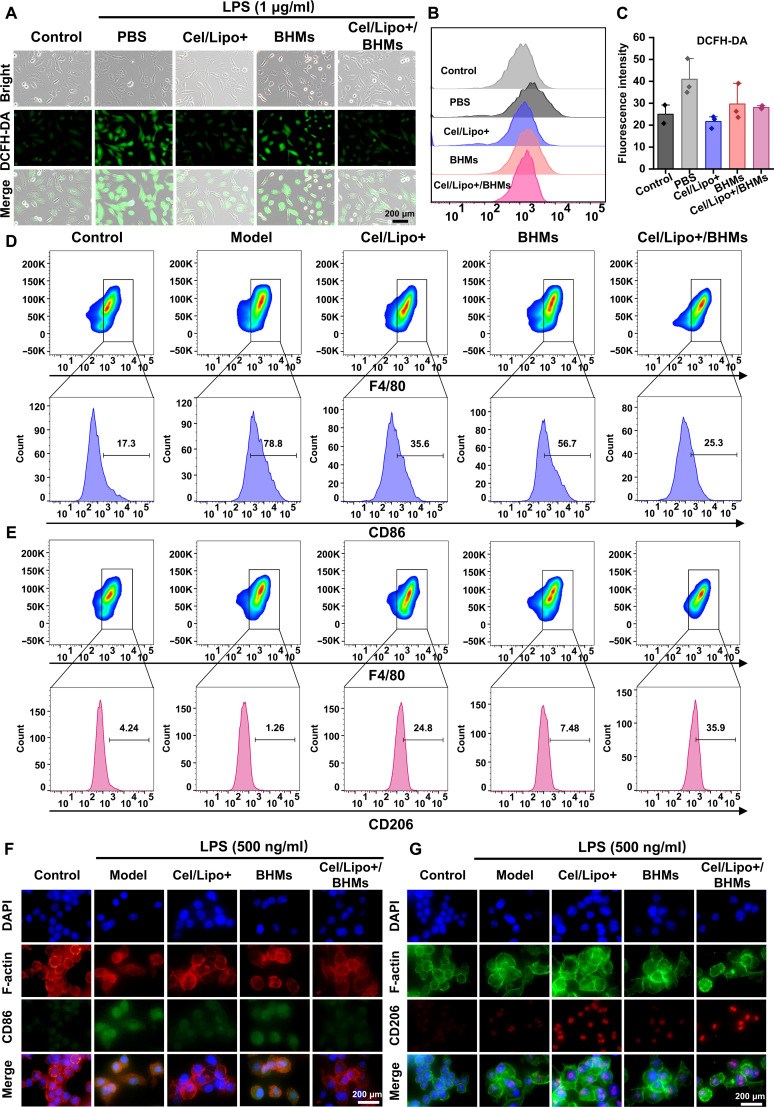
Cel/Lipo+/BHM microgels regulate chondrocyte oxidative stress and macrophage polarization in vitro. (A) Fluorescence microscope images of C28/I2 cells stained with DCFH-DA after different treatments. Scale bar, 200 μm. (B) Flow cytometry was used to analyze the fluorescence intensity of DCFH-DA in C28/I2 cells after different treatments. (C) Quantitative statistical analysis of DCFH-DA fluorescence intensity. (D and E) Flow cytometry was used to analyze the expression of RAW264.7 cells stained with anti-CD86 and anti-CD206 antibodies after different treatments. (F and G) The results of immunofluorescence showed the expression levels of CD86 and CD206. Scale bars, 200 μm. Data are presented as means ± SD. *n* ≥ 3.

Normal chondrocytes exhibit minimal amounts of ROS, whereas patients with OA demonstrate elevated ROS production and oxidative stress [59]. Concurrently, ROS stimulates the NF-κB signaling pathway, enhances the expression of MMPs such as MMP-13, and facilitates the secretion of inflammatory mediators such as IL-1β and TNF-α, leading to the expedited degradation of Col2 and proteoglycans, thereby establishing a detrimental cycle of ECM metabolism. Prior research has demonstrated that Cel and cationic liposome Cel/Lipo+ encapsulating Cel had remarkable ROS scavenging capabilities and can significantly mitigate oxidative stress damage in chondrocytes. We also assessed the capacity of BHMs and Cel/Lipo+/BHMs to modulate oxidative stress in joints. C28/I2 cells that received no treatment served as the negative control group. Other groups used LPS to activate C28/I2 cells, mimicking the aberrant expression of ROS in chondrocytes, while the positive control group utilized PBS for intervention. DCFH-DA is a fluorescent probe commonly used to detect intracellular ROS, efficiently indicating the intracellular ROS levels. ROS formation in C28/I2 cells was identified using immunofluorescence microscopy. The fluorescence staining results indicated that the fluorescence intensity of C28/I2 cells treated just with PBS was markedly greater than that of the other groups (Fig. 5A). The fluorescence intensity of the Cel/Lipo+ and Cel/Lipo+/BHM groups was markedly lower than that of the PBS group, indicating that Cel/Lipo+/BHMs successfully diminished ROS levels in C28/I2 cells. In addition, we used flow cytometry to quantitatively assess the ROS levels across several treatment groups. The findings indicated that the Cel/Lipo+ and Cel/Lipo+/BHM groups markedly diminished the expression of ROS (Fig. 5B and C), corroborating the outcomes of fluorescence labeling.

In the articular cavity microenvironment, DAMPs, including mechanical stress and cellular debris, induce macrophages to polarize toward the proinflammatory M1 phenotype through activation [15]. The secreted TNF-α and IL-1β not only augment ROS production in chondrocytes via paracrine signaling but also provoke the inflammatory transformation of synovial fibroblasts, leading to increased chemokine production, including C–C motif chemokine ligand 2 and C–X–C motif chemokine ligand 8, which recruit and further polarize macrophages, thereby establishing a positive feedback loop. The quantity of M2 macrophages with reparative functions is markedly diminished because of metabolic reprogramming disorders, leading to inadequate production of anti-inflammatory substances, which hinders the effective clearance of apoptotic debris and the initiation of tissue remodeling. A multidimensional pathogenic cascade characterized by oxidative stress, chronic inflammation, matrix degradation, and regeneration disorder is established, resulting in persistent suppression of joint repair. Consequently, the reprogramming of macrophages and the remodeling of the immunological milieu within the joint cavity are crucial for the healing of arthritis. A substantial body of literature has documented the regulatory influence of Cel on macrophages, demonstrating its capacity to inhibit M1 macrophage polarization and modulate macrophage polarization toward M2 via multiple pathways, thereby exhibiting pronounced anti-inflammatory and immunomodulatory effects. The distinctive mannose structure of BSP can efficiently bind to the mannose receptor on macrophage surfaces, suppress M1 proinflammatory polarization, and facilitate M2 reparative polarization. The Cel/Lipo+/BHMs created in this study successfully facilitated the synergistic accumulation of Cel and BSP in the inflammatory joint cavity via cationic-liposome-mediated transport and sustained local release from microgels. To clarify the potential macrophage reprogramming effect of the Cel/Lipo+/BHM system, we treated RAW264.7 cells with Cel/Lipo+, BHMs, and Cel/Lipo+/BHMs. F4/80 is a highly specific surface marker for macrophages, utilized to identify mature macrophages, while CD86 and CD206 serve as distinguishing markers for M1 and M2 macrophages, respectively. Flow cytometry analysis (Fig. 5D and Fig. S5A) demonstrated that CD86 expression in RAW264.7 cells attained 78.8% upon LPS stimulation, signifying a pronounced polarization of M0 macrophages to M1. The costimulation of LPS and Cel/Lipo+, as well as Cel/Lipo+/BHMs, significantly mitigated the expression of CD86. Simultaneously, as a result of the BSP effect, the expression of CD86 in Cel/Lipo+/BHMs was diminished compared to that in Cel/Lipo+ alone. This demonstrated that Cel/Lipo+/BHMs suppressed M1 macrophage polarization. Following treatment of RAW264.7 with Cel/Lipo+, BHMs, and Cel/Lipo+/BHMs, the expression of the M2 macrophage marker CD206 was elevated in both Cel/Lipo+ and Cel/Lipo+/BHMs, signifying an increase in M2 macrophage polarization (Fig. 5E and Fig. S5B). Furthermore, we validated the reprogramming action of macrophages, specifically the Cel/Lipo+/BHM system, using immunofluorescence labeling. The immunofluorescence intensity of CD86 was markedly increased with LPS stimulation alone; however, the fluorescence intensity of the CD86 marker diminished with the costimulation of LPS and Cel/Lipo+/BHMs (Fig. 5F and Fig. S6A). Simultaneously, the detection of the M2 macrophage marker CD206 revealed that the fluorescence intensity in the Cel/Lipo+ and Cel/Lipo+/BHM intervention groups surpassed that of the other treatment groups (Fig. 5G and Fig. S6B), corroborating the results obtained from flow cytometry. The aforementioned data demonstrate that Cel and BSP function synergistically within the Cel/Lipo+/BHM system, together facilitating the efficient reprogramming of macrophages from the proinflammatory M1 phenotype to the anti-inflammatory/repair M2 phenotype. The effective alteration of macrophage phenotype will further influence the profound restructuring of the articular immune milieu from a proinflammatory state to a state of repair homeostasis. Simultaneously, it will effectively suppress the synthesis of synovial inflammation and cartilage matrix degradation enzymes, while expediting the repair process of OA.

### Cel/Lipo+/BHM microgels regulate macrophage polarization by inhibiting the activation of NF-κB and TNF signaling pathways

**Fig. 6.**
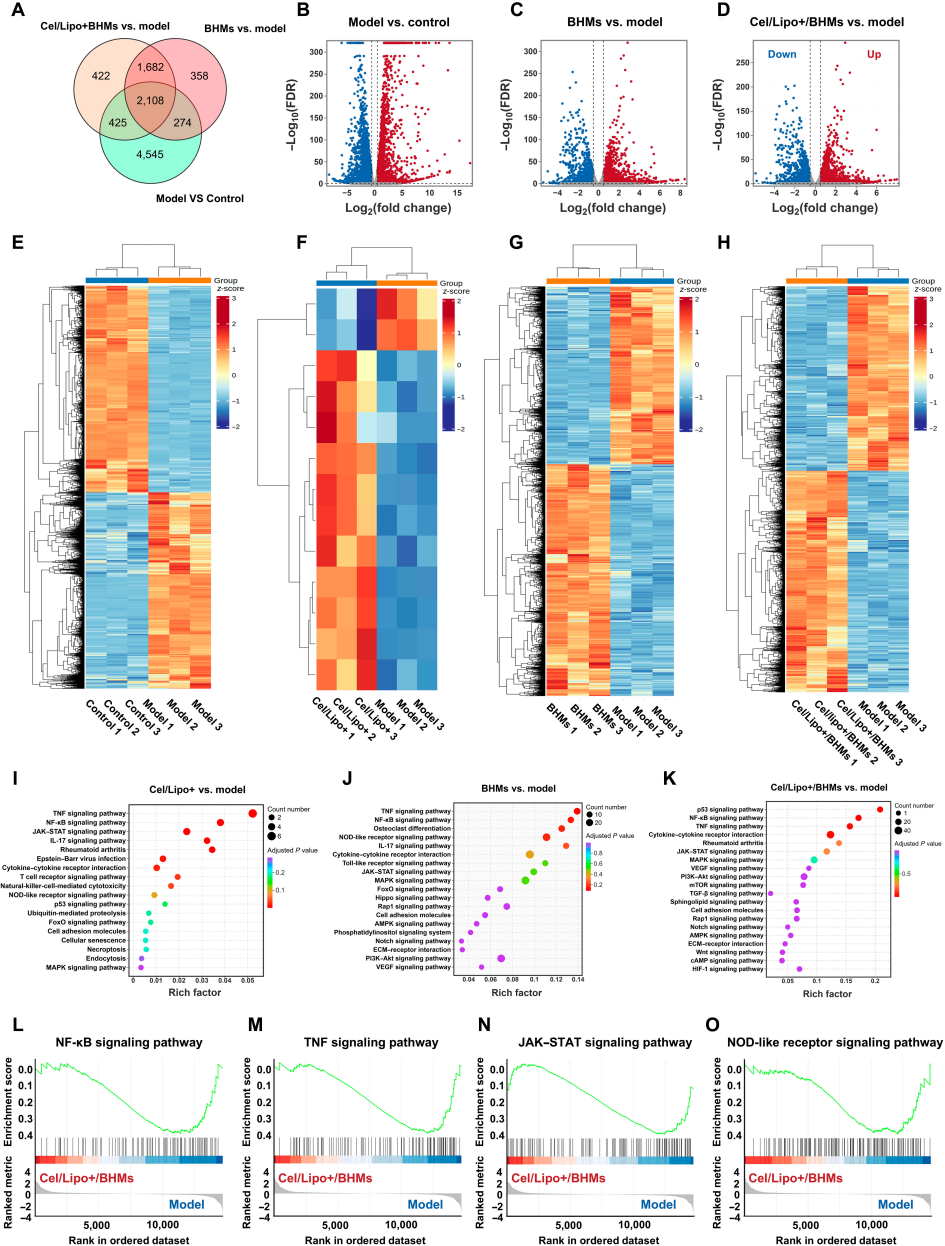
RNA sequencing analysis of macrophages subjected to different treatments. (A) Venn diagram of DEGs across different treatment groups (*n* = 3). (B) Volcano plot identifying up-regulated and down-regulated genes in the LPS-only treated model group. FDR, false discovery rate. (C) Volcano plot identifying up-regulated and down-regulated genes treated with BHM microgel. (D) Volcano plot identifying up-regulated and down-regulated genes treated with Cel/Lipo+/BHM microgel. (E) Heatmap analysis of cluster DEGs in untreated (control group) and LPS-treated (model group) RAW264.7 cells. (F) Heatmap analysis of cluster DEGs between Cel/Lipo+-treated and model group RAW264.7 cells. (G) Heatmap analysis of cluster DEGs between BHM-treated and the model group RAW264.7 cells. (H) Heatmap analysis of cluster DEGs between Cel/Lipo+/BHM-treated and LPS-treated model group RAW264.7 cells. (I) KEGG enrichment analysis for Cel/Lipo+ treatment. (J) KEGG enrichment analysis for BHM treatment. (K) KEGG enrichment analysis for Cel/Lipo+/BHM treatment.(L to O) GSEA performed on gene sets for the NF-κB, TNF, JAK–STAT, and nonobese diabetic (NOD)-like receptor signaling pathways.

To clarify the fundamental mechanisms of macrophage reprogramming induced by Cel/Lipo+/BHMs, we conducted whole-genome transcriptome sequencing analysis on macrophage models stimulated with various formulation groups (control group, model group, Cel/Lipo+ group, BHM group, and Cel/Lipo+/BHM triple combination group). Venn diagrams of differentially expressed genes (DEGs) (Fig. 6A), volcano plots (Fig. 6B to D), and heatmaps of gene expression demonstrated substantial disparities among the groups (Fig. 6E to H). In comparison to the model group, the BHM group displayed 4,422 DEGs (2,503 up-regulated and 1,920 down-regulated), whereas the Cel/Lipo+/BHM group revealed 4,637 DEGs (2,531 up-regulated and 2,106 down-regulated) in relation to the model group. Kyoto Encyclopedia of Genes and Genomes (KEGG) enrichment analysis revealed that TNF and NF-κB signaling pathways were consistently enriched in comparisons of Cel/Lipo+ against model, BHMs versus model, and Cel/Lipo+/BHMs versus model, indicating their role in M2 macrophage polarization produced by Cel/Lipo+/BHMs. The Janus kinase (JAK)–signal transducers and activators of transcription (STAT) signaling pathway was notably enriched in the comparisons of Cel/Lipo+ versus model and Cel/Lipo+/BHMs versus model, but not in BHMs alone, indicating that Cel modulates macrophage polarization through the JAK–STAT pathway (Fig. 6I to K). Moreover, gene set enrichment analysis (GSEA) results substantiated that the BHM treatment group markedly inhibited the TNF and NF-κB signaling pathways, thereby validating at the genomic level that BSP has distinctive immunomodulatory activity directed at innate immunity pathways. The Cel/Lipo+/BHM triple combination exhibited a synergistic inhibitory impact, simultaneously attenuating the TNF, NF-κB, and JAK–STAT signaling pathways (Fig. 6L to O). Simultaneously, quantitative polymerase chain reaction (qPCR) analysis indicated that Cel/Lipo+/BHMs inhibited the expression of proinflammatory genes (*TNF-α* and *Nos2*) while augmenting the expression of anti-inflammatory factors (*IL-10*) (Fig. S7). These findings collectively demonstrate that Cel/Lipo+/BHMs facilitate the phenotypic transition from M1 to M2 macrophages by suppressing the activation of TNF, NF-κB, and JAK–STAT signaling pathways, thereby restoring immune homeostasis and effectively alleviating damage from LPS-induced inflammatory microenvironments.

### Inhibition of persistent inflammation mediates chondrocyte function degradation

**Fig. 7.**
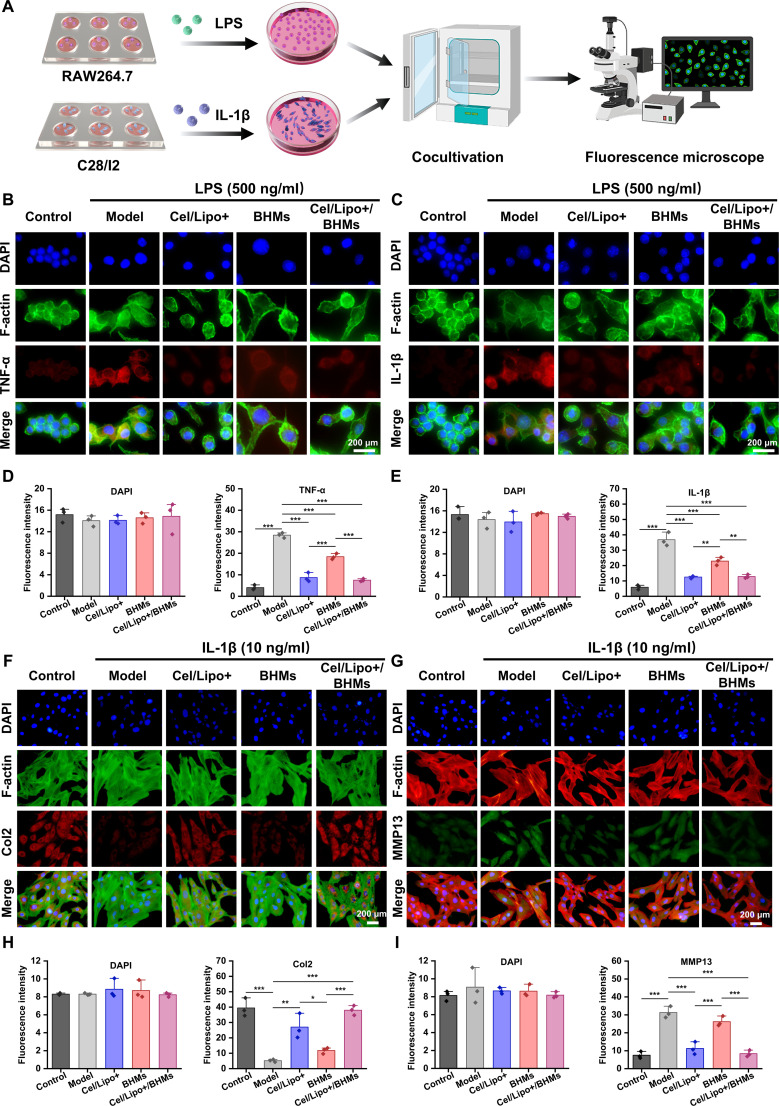
Cel/Lipo+/BHMs inhibit cartilage degeneration mediated by the inflammatory response. (A) Cell experiment process diagram. (B and C) Immunofluorescence images of TNF-α and IL-1β expression in RAW264.7 cells treated with LPS and different samples for 24 h. Scale bars, 200 μm. (D and E) Quantitative statistical analysis of immunofluorescence intensity of DAPI, TNF-α, and IL-1β. (F and G) Immunofluorescence images of Col2 and MMP13 expression in C28/I2 cells treated with IL-1β and different samples for 24 h. Scale bars, 200 μm. (H and I) Quantitative statistical analysis of immunofluorescence intensity of DAPI, Col2, and MMP13. Data were expressed as means ± SD. *n* ≥ 3. *P < 0.05, **P < 0.01, and ***P < 0.001.

The Cel/Lipo+/BHM microgel has shown a remarkable capacity to modify the immunological milieu within the joint cavity. Cel and BSP together facilitate the substantial reprogramming of macrophages from proinflammatory M1 to reparative M2. This reprogramming impact is not a singular occurrence. This reprogramming may result in the suppression of proinflammatory factors IL-1β and TNF-α production. The restructured anti-inflammatory immune microenvironment efficiently obstructs the deleterious paracrine influence of chronic inflammatory signals on chondrocytes, hence further impeding the degeneration of chondrocytes induced by sustained inflammation. Immunofluorescence investigation demonstrated that in the in vitro coculture model (Fig. 7A), the fluorescence intensity of TNF-α and IL-1β in RAW264.7 cells following Cel/Lipo+/BHM microgel intervention was markedly diminished (Fig. 7B and C). In the quantitative statistical analysis of fluorescence intensity, the expression levels of TNF-α and IL-1β in Cel/Lipo+/BHMs were 7.49 and 12.86, respectively, significantly lower than the expression levels of 28.39 and 36.82 seen in the model group (Fig. 7D and E). The expression of proinflammatory cytokines was markedly diminished. To investigate the potential impact of diminishing proinflammatory cytokine expression on chondrocyte functionality, we utilized the inflammatory factor IL-1β to activate C28/I2 chondrocytes, thereby simulating the OA cell model, and assessed the expression levels of Col2 and MMP13 in C28/I2 cells. Col2 is the predominant structural macromolecule in the ECM of articular cartilage. It is primarily produced and secreted by chondrocytes, imparting essential tensile strength and mechanical stability to cartilage tissue. In the pathological progression of OA, a sustained inflammatory milieu characterized by elevated levels of IL-1β and TNF-α markedly impairs the anabolic activity of chondrocytes, leading to reduced expression of Col2 and diminished chondrocyte functionality. MMP13, a significant member of the collagenase family, is regarded as the principal proteolytic enzyme responsible for the breakdown of the cartilage collagen network in OA pathology, owing to its high specificity and cleavage efficiency for Col2. In the inflammatory milieu of OA, chondrocytes are markedly activated by proinflammatory stimuli, resulting in a substantial up-regulation of MMP13 expression and activity, which causes the depolymerization and breakage of collagen fibers. The breakdown products of MMP13 concurrently activate the inflammatory signaling system, creating a detrimental loop that exacerbates the disintegration of the cartilage matrix and the apoptosis of chondrocytes. In the in vitro OA cell model, we used PBS, Cel/Lipo+, BHMs, and Cel/Lipo+/BHMs for intervention treatment, while establishing a blank control group. Immunofluorescence microscopy results indicated that (Fig. 7F and H) the expression of Col2 in the Cel/Lipo+ and Cel/Lipo+/BHM groups was significantly elevated, with the Cel/Lipo+/BHM group exhibiting a higher expression of Col2 than the Cel/Lipo+ group. This enhancement was attributed to the immune remodeling effects of Cel and BSP, which diminished the secretion of proinflammatory cytokines. The expression of Col2 was elevated. MMP13 immunofluorescence labeling revealed a significant reduction in MMP13 expression in the Cel/Lipo+ and Cel/Lipo+/BHM treatment groups (Fig. 7G). The quantitative statistical fluorescence intensity revealed that the expression of Cel/Lipo+/BHMs was inferior to that of Cel/Lipo+ (Fig. 7I), suggesting that the synergistic effect of Cel and BSP inhibited the aberrant expression of MMP13 induced by inflammatory stimuli. In summary, the Cel/Lipo+/BHM system restores immunological homeostasis in OA, diminishes the expression of proinflammatory cytokines, and inhibits the degradation of chondrocyte anabolic activity induced by chronic inflammation via macrophage reprogramming.

To validate the interaction sequence of “macrophage reprogramming–inflammation inhibition–cartilage repair”, we developed a chondrocyte model within an IL-1β inflammatory microenvironment. Following the administration of M2 macrophage conditioned medium induced by Cel/Lipo+, BHM, and Cel/Lipo+/BHM, qPCR and immunofluorescence analyses revealed that Cel/Lipo+/BHM treatment markedly up-regulated the expression of the Col2 gene, a fundamental marker of cartilage synthesis, while effectively suppressing the expression level of the matrix-degrading enzyme MMP13 (Fig. S8A to C). Regulating macrophage polarization efficiently inhibits joint inflammation and promotes cartilage healing. Simultaneously, we used the Transwell 2-layer model to replicate the milieu of cartilage injury and observed that the quantity of M2 macrophages moving to the injured region was markedly greater than that in the model group (Fig. S8D). The data indicate that Cel/Lipo+/BHM-induced M2 macrophages not only alter the anabolic equilibrium of chondrocytes through factor secretion but also actively migrate to the cartilage injury site to establish a localized repair microenvironment, thereby creating a closed-loop mechanism from immune reprogramming to cartilage repair.

### Cel/Lipo+/BHM articular cavity distribution and continuous drug therapy

**Fig. 8.**
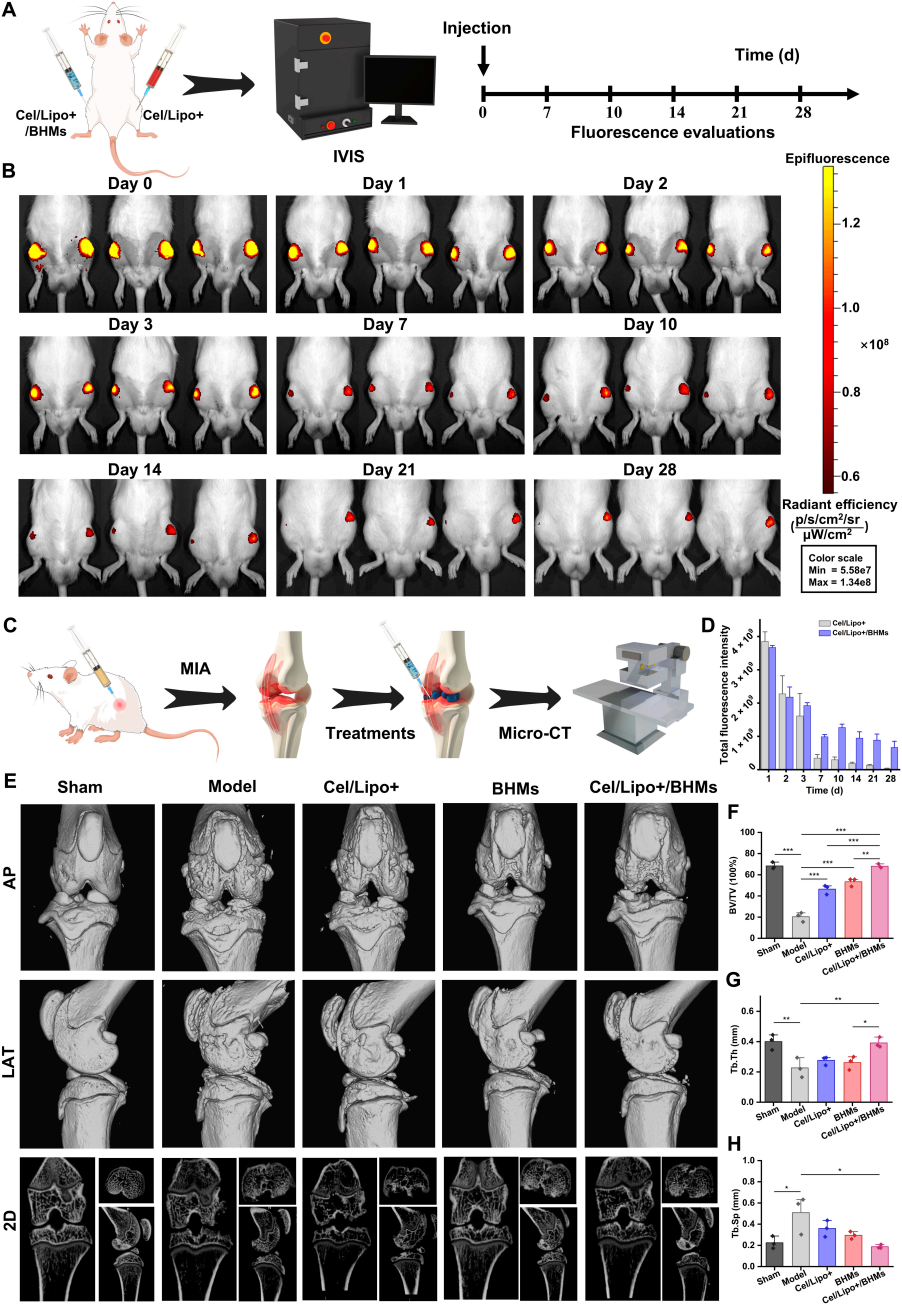
Micro-CT analysis of Cel/Lipo+/BHM microgel articular cavity sustained drug retention and in vivo treatment of the OA model. (A) The flow chart of Cel/Lipo+ and Cel/Lipo+/ BHM joint retention experiments was analyzed using in vivo imaging system (IVIS) imaging technology. (B) Representative images of time-dependent retention of Cel/Lipo+ and Cel/Lipo+/BHMs in rat articular cavities. (C) Overview of the process diagram of rats undergoing MIA-induced OA model, intervention, and imaging. (D) Quantitative analysis of the fluorescence intensity of Cel/Lipo+ and Cel/Lipo+/BHMs. (E) Representative micro-CT images and longitudinal 2-dimensional (2D) micro-CT sections in anteroposterior (AP) and lateral (LAT) views of knee joints of OA rats after treatment. (F to H) Quantitative analysis of bone volume ratio (BV/TV), trabecular thickness (Tb.Th), and trabecular separation (Tb.Sp). All data are expressed as means ± SD. *n* = 5. *P < 0.05, **P < 0.01, and ***P < 0.001.

To assess the targeted delivery efficacy and sustained therapeutic potential of Cel/Lipo+/BHM composite microgels in the articular cavity of OA, we systematically examined the spatiotemporal distribution and retention properties of Cel/Lipo+/BHM microgels in the joint using in vivo imaging technology before treatment (Fig. 8A). The results indicated that Cel/Lipo+/BHMs had a significantly greater retention capacity compared to Cel/Lipo+. Following intra-articular injection, Cel/Lipo+ and Cel/Lipo+/BHMs were localized within the joints. Following the 7th day, the fluorescence signal of Cel/Lipo+ exhibited a marked decrease, whereas the fluorescence of Cel/Lipo+/BHMs was significantly elevated compared to Cel/Lipo+ (Fig. 8B), underscoring the delivery benefits of the BHM-mediated microgel system. Significantly, Cel/Lipo+/BHMs have a distinctive functional accumulation within the joint. Microgels preferentially adhere to the cartilage surface in load-bearing regions due to their biomimetic lubrication properties, creating a repository for sustained drug release. Conversely, the cationic liposome Cel/Lipo+, released through the friction and wear of Cel/Lipo+/BHM microgels, effectively targets and persists in the microenvironment surrounding negatively charged chondrocytes, thereby exerting a regulatory effect. The retention kinetics investigation showed that the drug retention period in the joint cavity mediated by Cel/Lipo+/BHM microgel was considerably extended to 28 d (Fig. 8D), surpassing that of Cel/Lipo+. This particular distribution and prolonged retention mode within the joint cavity not only ensures a continuous foundation for Cel/Lipo+/BHM biolubrication but also guarantees the synergistic effect of the various biological activities of Cel and BSP at the lesion site.

### Efficacy evaluation of OA in vivo

To assess the synergistic therapeutic potential of Cel/Lipo+/BHM microgels in a complicated pathological environment in vivo, we used a monosodium iodoacetate (MIA)-induced Sprague–Dawley rat OA model. The MIA-induced model rapidly elicits a robust synovial inflammatory response, increasing degradation of the cartilage matrix and aberrant remodeling of the subchondral bone, closely mimicking the principal clinical characteristics of acute progressive OA. Following successful modeling, the rats were randomly allocated into 5 groups: sham operation control group (sham; administered normal saline only), model control group (model; MIA + normal saline), cationic liposome treatment group (Cel/Lipo+; MIA + Cel/Lipo+), blank microgel group (BHMs; MIA + BHMs), and composite microgel group (Cel/Lipo+/BHMs; MIA + Cel/Lipo+/BHMs). Periodic treatment via intra-articular injection aims to assess its long-term therapeutic impact, predicated on the superior distribution and retention properties observed in the initial phase. Joint tissue samples were taken after the treatment (Fig. 8C). Micro-computed tomography (CT) 3-dimensional reconstruction and quantitative analysis demonstrated substantial disparities in the microstructure of the subchondral bone between the groups. The model exhibited characteristic features of OA bone lesions, including extensive osteophyte formation and destruction of the subchondral bone plate, alongside disintegration of the trabecular structure (Fig. 8E). This was evidenced by a marked reduction in bone volume fraction, which was 47.96% lower than that of the control group, a pronounced decrease in trabecular thickness, and a significant increase in trabecular separation (Fig. 8F to H). Importantly, the Cel/Lipo+/BHM treatment demonstrated comprehensive bone protection beyond a singular component, substantially reversing the aberrant decrease in BV/TV and markedly restoring the trabecular microarchitecture by synergistic regulation. The trabecular thickness reverted to the level of the sham group, and the pathological breadth of the trabecular space was significantly inhibited. Its efficacy was markedly superior to that of the BHM group and the Cel/Lipo+ group, which solely offered mechanical lubrication. This outcome demonstrates that the Cel/Lipo+/BHM system can proficiently inhibit the pathological sclerosis of the subchondral bone and the deterioration of the trabecular network during OA through multifaceted intervention. This efficacy is attributed to the lubrication and drag reduction properties of BHMs, the prolonged retention characteristics of the drugs, the targeted antagonism of Cel on chondrocyte oxidative stress, and the synergistic modulation of Cel/BSP on the inflammatory pathway of synovial macrophages.

### Immunohistochemical analysis of joint tissue

**Fig. 9.**
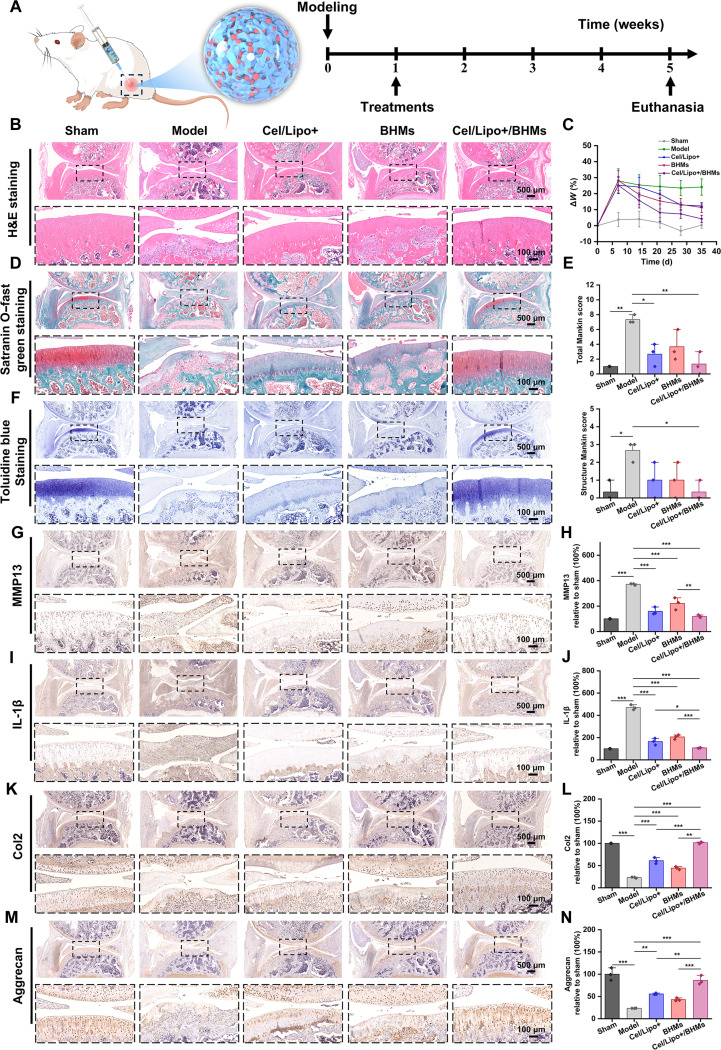
Histological evaluation of Cel/Lipo+/BHM microgels promoting cartilage matrix regeneration in rats. (A) Treatment schematic and intervention time node diagram of the OA rat model. (B, D, and F) Representative pictures of knee joint H&E, safranin O–fast green staining, and toluidine blue staining in OA rats. Scale bars, 500 and 100 μm. (C) Quantitative analysis of joint swelling in OA rats at different time points. (E) Total and structural Mankin scores of knee joints of OA rats in different treatment groups were calculated according to histological staining. (G, I, K, and M) The representative images of MMP13, IL-1β, Col2, and aggrecan protein immunohistochemical staining in the subchondral bone of the rat knee joint after treatment. Scale bars, 500 and 100 μm. (H, J, L, and N) Articular cartilage in the subchondral bone relative to MMP13, IL-1β, Col2, and aggrecan expression, with a positive area of quantitative. All data are expressed as means ± SD. *n* = 5. *P < 0.05, **P < 0.01, and ***P < 0.001.

To further investigate the multipathway reversal effect of the Cel/Lipo+/BHM system on the principal pathological processes of OA (cartilage degradation, synovial inflammatory infiltration, and matrix synthesis inhibition), we assessed the histopathological and molecular phenotype of the joint tissues in OA model animals posttreatment at the histological level (Fig. 9A). The impact of Cel/Lipo+/BHM microgels on the regeneration of the cartilage ECM was assessed by hematoxylin and eosin (H&E) staining, safranin O–fast green staining, and toluidine blue staining. The model group exhibited characteristic OA cartilage deterioration, encompassing surface erosion, chondrocyte clustering, elimination of the tidal mark, and a progressive reduction of proteoglycan (shown by saffron solid green and toluidine blue staining depth) alongside disorganization of the collagen network structure (Fig. 9B, D, and F). The Cel/Lipo+/BHM treatment group exhibited the most pronounced restoration of cartilage structure, evidenced by a relatively intact cartilage surface and organized cellular arrangement. Notably, there was a significant recovery in the depth and extent of safranine solid green and toluidine blue staining, which validated the effective supplementation and preservation of proteoglycan and glycosaminoglycan content within the cartilage matrix. The degree of joint swelling and modified Mankin score were used to evaluate the degree of articular cartilage injury. The swelling degree and Mankin scores of Cel/Lipo+/BHM group were significantly lower than those of the model group, Cel/Lipo+ group, and BHM group, suggesting that Cel/Lipo+/BHMs could alleviate OA cartilage injury (Fig. 9C and E). The expression of critical proinflammatory factors TNF-α and IL-1β in the articular cartilage and synovial tissue of the Cel/Lipo+/BHM group was significantly and synergistically suppressed (Fig. 9I and J and Fig. S9A and B), aligning closely with the in vitro efficacy of Cel and BSP in modulating macrophages and diminishing the secretion of inflammatory factors. This suggests that the composite system effectively restructured the inflammatory microenvironment of the joint cavity in vivo. Concurrently, the expression of MMP13, a principal mediator of cartilage degradation, was markedly down-regulated in the Cel/Lipo+/BHM group (Fig. 9G and H), whereas the synthesis and deposition of Col2 and aggrecan, the primary structural components of the cartilage matrix, were significantly enhanced (Fig. 9K to N). The suppression of MMP13 degradation activity and the promotion of Col2/aggrecan anabolism collectively form a dual molecular barrier for Cel/Lipo+/BHMs, preserving cartilage matrix homeostasis and countering degeneration, with their synergistic effect markedly superior to that of individual component treatment. The synergy of the Cel component’s antioxidative and anti-inflammatory properties, alongside the mechanical lubrication and prolonged drug retention capabilities offered by BHMs, may constitute the fundamental mechanism underlying this cartilage protective effect. In conclusion, the outcomes of in vivo experiments consistently indicated that Cel/Lipo+/BHMs facilitated a multifaceted and multichannel effective treatment of OA joints by synergistically modulating the inflammatory response (inhibiting TNF-α and IL-1β), suppressing matrix degradation (reducing MMP13), enhancing matrix synthesis (increasing Col2 and aggrecan), and ameliorating subchondral bone structure. The remarkable efficacy results from the distinctive benefits and synergy of Cel’s active components and the BHM carrier system in anti-inflammatory, antioxidant, matrix metabolism management, and sustained administration.

### Biosafety and immunogenicity evaluation of Cel/Lipo+/BHM microgels in vivo

We specifically developed a 30-d repeated dose paradigm in Sprague–Dawley rats. Blood biochemical parameters indicated that all hepatotoxicity markers (alanine aminotransferase, aspartate aminotransferase, and alkaline phosphatase) and nephrotoxicity indicators (creatinine, urea, and uric acid) were maintained within normal physiological limits across all groups (Fig. S10A to G), exhibiting no statistically significant difference when compared to the saline control group. Upon euthanasia at the conclusion of the study, histological examination of the heart, liver, spleen, lungs, and kidneys was conducted. Following fixation in 4% paraformaldehyde, paraffin embedding, and H&E staining, all organs displayed no pathological changes (Fig. S10H). The liver exhibited preserved hepatic lobule structure with radially organized hepatocyte cords, lacking steatosis or necrotic areas. The renal glomeruli had normal shape without thickening of Bowman’s capsule, and the renal tubular epithelial cells displayed no edema or cast formation. Heart, spleen, and lung tissues also exhibited no evidence of inflammatory infiltration or fibrosis. In the injected knee joint, synovial tissue exhibited no infiltration of lymphocytes or plasma cells nor any foreign-body giant cell reactions, while the articular cartilage surfaces remained intact and smooth, indicating that the microgel material did not provoke local immune rejection or tissue injury. The findings collectively demonstrate that the BSP/HA dual-cross-linked microgel markedly diminished systemic exposure to Cel via sustained release kinetics, while the naturally sourced carrier materials (HA and BSP) exhibited exceptional biocompatibility, as prolonged administration did not elicit hepatorenal damage or systemic/local immune responses. To evaluate the influence of microgel degradation products on the local joint immune microenvironment, multiplex immunohistochemical staining of knee joint tissues from the repeated-dosing model demonstrated that CD68^+^ macrophages were sparsely distributed within the synovial sublayer, lacking aggregation. The expression of myeloperoxidase-positive (MPO^+^), signifying neutrophil infiltration, was modest, and C3d^+^ deposition, a crucial indicator of complement activation, was absent in the cartilage–synovium junctional region, aligning with the native joint condition (Fig. S11). The data indicate that the breakdown products of the microgel did not elicit aberrant immune cell recruitment, persistent inflammation, or activation of the complement cascade. The advantageous metabolic compatibility arises from the intrinsic characteristics of the materials, therefore maintaining joint immunological homeostasis.

## Conclusion

This study addresses the inherent contradiction between the remarkable anti-inflammatory and antioxidant properties of Cel and its systemic toxicity, alongside the distinctive pathological features of oxidative stress, chronic inflammation, negative electrical barriers, and mechanical degradation within the OA joint cavity, by innovatively developing a “trinity” synergistic delivery strategy. Initially, the “attenuation and enhancement” of Cel and the targeted delivery of chondrocytes were accomplished through cationic liposome encapsulation, utilizing electrostatic interactions to overcome the negative electrical barrier of joints, thereby ensuring precise drug delivery and markedly diminishing off-target toxicity. Second, BSP/HA double-cross-linked microgel BHMs exhibiting bionic bearing rolling lubrication properties were engineered, which not only significantly enhanced the biolubrication efficacy of the damaged joints but also proficiently encapsulated Cel/Lipo+ as a carrier to address the issue of the limited retention duration of conventional liposome joints. The in vivo distribution study confirmed its advantage in long-term retention. Ultimately, Cel and BSP were used to modulate the phenotypic reprogramming of macrophages, alter the immunological milieu of the joint cavity, and suppress the surge of proinflammatory cytokines TNF-α and IL-1β. In vivo investigations confirmed the remarkable efficiency of the microgel in the MIA-induced rat OA model. Cel/Lipo+/BHMs markedly suppressed the expression of the cartilage-matrix-degrading enzyme MMP13, enhanced the synthesis and deposition of Col2 and aggrecan, effectively reversed cartilage degeneration, and ameliorated abnormal subchondral bone remodeling through a multifaceted synergistic mechanism of lubrication-targeted delivery-immune regulation. Moreover, its efficacy surpasses that of any single-component treatment (Cel/Lipo+ or BHMs), underscoring the indispensable synergistic effects of prolonged carrier retention, biological lubrication, intracellular targeted delivery of active ingredients, and remodeling of the immune microenvironment. This study offers a novel solution for the clinical management of highly toxic natural products and introduces an innovative treatment concept that concurrently targets the multifaceted pathological processes of OA, including mechanical wear, oxidative stress, inflammatory cascades, and cartilage dysfunction, via a bionic delivery system. It also presents new insights and platforms for the advancement of OA therapeutic formulations.

## Materials and Methods

### Materials

Cel (purity > 98%) was purchased from Chengdu Desite Biological Technology Co. Ltd. (Chengdu, China). BSP was extracted using the existing methods in the laboratory. Egg yolk lecithin and cholesterol were purchased from AVT Pharmaceutical Technology Co. Ltd. (Shanghai, China). MA, 5-isothiocyanate fluorescein, and MIA were purchased from Shanghai Yansu Technology Co. Ltd. (Shanghai, China; mall.shiyanjia.com). LPS purchased from Beijing Solarbio Science & Technology Co. Ltd. Anti-TNF-α was purchased from Zen bioscience. Recombinant human IL-1β was purchased from Chamot Biotechnology Co. Ltd. Super fetal bovine serum was purchased from Procell Life Science & Technology Co. Ltd. Matrigel matrix was purchased from Corning Incorporated (NY, USA). MRC1/CD206 (DF4149) and IL-1β (AF5103) antibodies were purchased from Affinity Biosciences. Cell flow antibody allophycocyanin anti-mouse CD86 antibody, phycoerythrin anti-mouse CD206 (macrophage mannose receptor) were purchased from BioLegend (San Diego, USA). FITC anti-mouse F4/80 antibody (FMF480-02-100) was purchased from 4A Biotech. All other reagents were of analytical grade.

### Cell culture

The C28/I2 human normal cartilage cell line and RAW264.7 mouse mononuclear macrophage cell line were cultured in a 37 °C and 5% CO_2_ incubator. The basic medium was high-glucose Dulbecco’s modified Eagle’s medium (Gibco, catalog no. 11965092) containing 10% (v/v) fetal bovine serum (Gibco) and 1% penicillin–streptomycin (HyClone). The medium was replaced every 48 h. When the cell fusion reached 80%, 0.25% trypsin–EDTA (Biosharp) was used for digestion and passage. All cell lines were identified by short tandem repeat, and the number of passages was controlled between 5 and 15 generations.

### Animals

All rats were purchased from SPF Biotechnology Co. Ltd. (Beijing, China). The ambient temperature was 24 ± 2 °C, and the relative humidity was 55 ± 5%. During the experiment, the bed was changed once a day. According to the recommendations for the treatment and use of experimental animals issued by the National Institutes of Health, all animals were approved by the Experimental Animal Management Association of Chengdu University of Traditional Chinese Medicine (ethical batch number: 2024-029).

Methods are detailed in the Supplementary Materials.

## Data Availability

The data supporting the results of this study can be obtained from the corresponding authors according to reasonable requirements.
