# Supplementary material for: Microfluidic Nanocomposite Lubricating Microgels Localized Deliver Celastrol for Remodeling the Immune Microenvironment to Enhance Osteoarthritis Treatment
Source: Research (Wash D C). 2026 Feb 27;9:1124. doi: 10.34133/research.1124 (PMC12946385; doi:10.34133/research.1124)
Supplement: Supplementary 1 — Supplementary Methods Figs. S1 to S11 Table S1 [file research.1124.f1.docx]

**Supplementary Materials**

**Microfluidic Nanocomposite Lubricating Microgels Localized Deliver Celastrol for Remodeling the Immune Microenvironment to Enhance Osteoarthritis Treatment**

**Authors**

Peng Guo^1^, Qiaolin Yang^1^, Wen Shi^1^, Qin Yang^1^, Yuchun Liu^1^, Ya Tian^1^, Wenbi Tuo^1^, Xiaoli Yi^1^, Jun Zhao^1^, Siwei Xiong^1^, Weidong Zhang^1^, Rui Zeng ^2^, Chen Zhang^1*^, Yan Qu^1*^

**Affiliations**

1. Chinese Medicine Germplasm Resources Innovation and Effective Uses Key Laboratory of Sichuan Province, Chengdu University of Traditional Chinese Medicine, Chengdu, 611137, China

2. Key Laboratory of Research and Application of Ethnic Medicine Processing and Preparation on the Qinghai Tibet Plateau, Chengdu 610225, China

3. Sichuan Province Engineering Technology Research Center of Natural Small Molecule Drug, Tianfu TCM Innovation Harbour, Chengdu University of Traditional Chinese Medicine, Chengdu, 611930, China

*Address correspondence to: [chenzhang_1990@126.com(C.Z.)](mailto:chenzhang_1990@126.com(C.Z.)); [quyan028@cdutcm.cn](mailto:quyan028@cdutcm.cn)(Q.Y.)

**Supplementary Methods**

**Fabrication and Characterization of Cel/Lipo**

Cationic (Cel/Lipo+) and anionic (Cel/Lipo-) celastrol liposomes were synthesized using the film dispersion technique. 30 mg of egg yolk lecithin, 6 mg of cholesterol, 4 mg of stearylamine, and 2 milligrams of Cel were dissolved in 15 mL of chloroform to create a homogenous solution. The solvent was eliminated using rotary evaporation at 45°C to yield a lipid film, after which 6 mL of deionized water was introduced for hydration at 37°C for 30 minutes, followed by ultrasonic treatment for 5 minutes to produce Cel/Lipo+. Preparation of Cel/Lipo- except omitting stearic amine; all other stages remain unchanged.

The particle size distribution, polydispersity index (PDI), and zeta potential of Cel/Lipo were assessed by dynamic light scattering (DLS, Malvern Nano-ZS, UK) at 25°C following the dilution of Cel/Lipo. The variations in particle size were observed over 7 consecutive days to assess in vitro stability. The morphology of cationic and anionic Cel/Lipo was analyzed using transmission electron microscopy (TEM, FEL Tecnai G2 12). The diluted sample was applied to the copper mesh, and the surplus liquid was absorbed with filter paper after a 5-minute interval. A 3% phosphotungstic acid solution was negatively stained for 1 minute and examined under transmission electron microscopy upon drying.

**Toxicity determination of Cel**

To elucidate the cytotoxic effect of Cel and assess the mitigating effect of its liposomal delivery system, the CCK-8 technique was employed to evaluate the cytotoxicity of varying doses of free Cel and Cel/Lipo formulations. C28/I2 cells were inoculated in 96-well plates at a density of 1×10^4^ cells per well. Following overnight culture, the cells were subjected to treatment with free Cel, anionic Cel/Lipo-, and cationic Cel/Lipo+. The Cel concentration gradient was established at 0-10 μg/mL (0, 0.125, 0.25, 0.5, 1, 2.5, 5, 10 μg/mL). Following 24 hours of pharmacological intervention, cell viability was assessed using a CCK-8 kit, with absorbance recorded at 450 nm via a microplate reader (SpectraMax iD5).

**Cel/Lipo chondrocyte targeting analysis**

This study performed a systematic assessment of in vitro cell uptake studies to verify the cartilage-targeting characteristics of Cel/Lipo+. The C28/I2 cells were inoculated at a density of 5×10 per plate on a confocal culture dish. Following 24 hours of standard culture to confirm complete cell adherence, the fluorescent tracer coumarin 6 (C6) was encapsulated within the liposomal structure using a thin film hydration technique in accordance with the Cel/Lipo preparation protocol. C28/I2 cells were treated for 4 hours at 37°C with 5% CO_2_, using free C6 group, anionic Cel/Lipo- group, and cationic Cel/Lipo+ group. The subcellular localization was examined using a fluorescent microscope (ZEISS LSM 880) to acquire spatial distribution data of the fluorescence signal. The intensity of cell internalization was quantitatively assessed by flow cytometry (BD FACSVers). The original data were analyzed using FlowJo V10.8 software, and the mean fluorescence intensity, together with the percentage of positive cells in each group, was computed.

**Cel/Lipo chondrocyte ROS clearance**

The C28/I2 cell model was employed to assess the scavenging effect of Cel on intracellular ROS. The cells were inoculated in a confocal culture dish at a density of 5×10^4^ per dish and were routinely grown for 24 hours until complete adherence was achieved. The oxidative stress model was subsequently established through pretreatment with LPS (1 μg/mL). The experimental groups received Cel monomer and Cel/Lipo (both containing the identical dosage of Cel) for 6 hours. The DCFH-DA fluorescent probe method was employed to assess ROS levels: cells were incubated with 10 μM probe for 30 minutes in the dark, thereafter washed three times with PBS to eliminate leftover dyes, and DAPI nuclear staining (5 μg/mL, 3 min) was conducted for fluorescence microscopy (Ex/Em=488/525 nm). The intracellular fluorescence intensity was quantitatively measured by flow cytometry, and the rate of ROS removal was computed. All studies were conducted using three biological replicates.

**Synthesis and characterization of BSPMA and HAMA**

To prepare BSPMA, 200 mg of BSP was dissolved in 60 mL of deionized water, followed by the addition of 0.745 mL of methacrylic anhydride, while maintaining the pH of the reaction system at 8.0-8.5 using 4% (w/v) NaOH. The reaction was conducted in darkness at 25°C for 24 hours, followed by centrifugation at 8000 rpm for 15 minutes. The supernatant was purified using a 3.5 kDa dialysis bag for three days, and the product was acquired through freeze-drying. The synthesis of HAMA involved dissolving 1 g of HA in 100 mL of deionized water, followed by the gradual addition of 2 mL of methacrylic anhydride in an ice bath. A 4% NaOH solution was employed to maintain a pH of 8.0-8.5, and the reaction was conducted at 4°C for 24 hours. Following centrifugation and purification, it underwent dialysis and lyophilization under identical circumstances. The substitution degree of the two synthesized products was quantitatively assessed using 1H-NMR (Bruker AVANCE III, 600 MHz), and the grafting rate was determined from the integral area of the methacryloyl characteristic peak (δ5.6-6.1 ppm). FT-IR (Nicolet iS50, 4000-500 cm^-1^) was employed to analyze alterations in chemical bonds. BSPMA exhibited an ester carbonyl absorption peak at 1720 cm-1, while HAMA had a C=C double bond vibration peak at 1630 cm-1, thus validating the effective grafting of the methacryloyl group.

**Preparation and characterization of Cel/Lipo+/BHMs precursor gel**

To assess the gelation characteristics of the photocrosslinking system, specific concentrations of BSPMA, HAMA, and B-HMA precursor solutions were formulated. The photocuring characteristics were assessed using the Xilin bottle inversion method following the addition of 0.1% (w/v) photoinitiator LAP. A 2 mL slurry was poured into a bespoke cylindrical polytetrafluoroethylene mold (Φ10×5 mm) and treated with 405 nm ultraviolet radiation for 5 minutes to produce a solid hydrogel. The storage modulus (G') and loss modulus (G") were quantified using a rotational rheometer (Anton Paar, MCR72). Each sample group was measured in triplicate.

**Preparation and characterization of Cel/Lipo+/BHMs microgel**

This work employed microfluidic technology to develop a drug-loaded microgel device. The precise preparation procedure is as follows: The Cel/Lipo+ solution, comprising 1% BSPMA, 0.5% HAMA, and 0.5% photoinitiator LAP, served as the aqueous phase (50 mg BSPMA and 25 mg HAMA were dissolved in 5 mL Cel/Lipo+), while the oil phase was formulated from 95% liquid paraffin and 5% Span80. The oil phase flow rate (5 mL/h) and the water phase flow rate (0.4 mL/h) were precisely regulated by a dual-channel injection pump. The two phases were introduced into the primary channel of the microfluidic chip, where the water phase was segmented by the shear force of the two-phase fluid, resulting in the formation of monodisperse emulsion droplets. The droplets were gathered and exposed to a 405 nm UV light source (3.5 mW/cm²) for 5 minutes to achieve complete photocrosslinking. The acquired microgels were rinsed thrice with 75% ethanol and deionized water to eliminate surfactant and oil phase remnants, and thereafter kept in deionized water at 4°C.

Characterization of microgel properties: The macroscopic morphology of the microspheres was examined using an inverted optical microscope (IX73, Olympus). One hundred microspheres were randomly chosen for morphological study, and the particle size distribution was determined using ImageJ software. To confirm the uniformity of drug-loaded distribution, a FITC-labeled Cel/Lipo+ drug-loaded system and a rhodamine B-labeled microgel matrix network were employed for three-dimensional fluorescence co-localization assessment using laser confocal microscopy (Leica, TCS SP8). The samples underwent microstructure characterization by being frozen in liquid nitrogen, freeze-dried for 24 hours, affixed to conductive glue, and then coated with gold for 60 seconds at a current of 15 mA to improve conductivity. Secondary electron signals were collected using scanning electron microscopy (ZEISS, Sigma 300) at a 5 kV acceleration voltage to examine the surface topology and pore properties of the microspheres.

**Drug encapsulation and in vitro release**

An adequate volume of Cel/Lipo+ solution was filtered via a 0.22 μm microporous membrane, and 1 mL of the filtrate was precisely extracted, combined with 9 mL of methanol for dissolution and ultrasonic demulsification, after which the Cel content (Mpackage) was quantified. Furthermore, 1 mL of unfiltered Cel/Lipo+ solution was diluted in 9 mL of methanol, followed by ultrasonic demulsification to ascertain the total amount of Cel (Mtotal). The encapsulation efficiency (EE%) was determined as follows.

Encapsulation efficiency (%) = Mpackage/Mtotal×100 %

The in vitro release characteristics of free Cel, Cel/Lipo+, and Cel/Lipo+/BHMs were examined using the dialysis method. Five milliliters of the aforementioned sample solution, containing 200 μg/mL Cel, were placed in a dialysis bag with a molecular weight cut-off of 10 kDa and immersed in 30 milliliters of release medium (phosphate buffer solution containing 30% (w/w) methanol). All samples were incubated at 37°C in a thermostatic oscillator set to 100 rpm. At the specified time intervals (0.5, 1, 2, 4, 6, 8, 12, 24, 48, 72, 96 hours), 1 mL of release medium was extracted and immediately supplemented with an equivalent volume of isothermal fresh medium, with three replicates established for each experimental group. The absorbance of Cel in the release media at each time point was quantified using a microplate reader, and its concentration was determined. The cumulative release percentage (Qn%) of Cel at time point n was determined using the subsequent formula. The drug release curve was constructed using time on the x-axis and cumulative release % on the y-axis.

Qn%=(Cn×V)+Σ(Ci×Vi)]/M×100%

In the formula, Q% is the cumulative release percentage of the drug at the nth time point; Cn is the concentration of each Cel calculated by the nth sampling; V is the total volume of dialysis medium in the centrifuge tube; Vi is the volume of each sampling at different time points; M is the quality of the dialysis bag containing Cel.

**Cel/Lipo+/BHMs microgel friction performance testing**

The reciprocating sliding friction was assessed using a multipurpose friction and wear tester (UMT-2, Bruker). The experimental setup employed a pin-disk contact mode, utilizing a Ti6Al4V titanium alloy disk (lower sample) with a surface roughness of Ra = 0.05 μm as the substrate, and a PTFE cylindrical pin with a diameter of 8 mm (effective contact surface diameter of 5 mm, Ra = 0.8 μm) as the upper friction component. The reciprocating sliding amplitude of 4 mm and a frequency of 1 Hz were established to replicate the joint motion period. The peak pressure in the contact area was determined to be 25.68 MPa under a 1 N normal load, as per Hertz contact theory, which encompasses the physiological load levels of human joints. Before the experiment, 20 μL of the lubricant under investigation (PBS solution with 5 mg/mL microgel) was pre-applied to the contact region, while PBS solution served as the negative control. Each experimental group had a 60-minute dynamic friction test, during which the friction coefficient variation was recorded in real-time by the integrated three-dimensional force sensor. To guarantee the experiment's reproducibility, all test conditions were independently replicated three times (n=3), and the mean friction coefficient during the steady phase (20-50 min) was utilized for statistical analysis.

**In vitro swelling and degradation analysis**

Determination of swelling rate: 10 mg of freeze-dried BHMs and Cel/Lipo+/BHMs microgels were submerged in 4 mL of deionized water and agitated at 37°C and 80 rpm. Samples were collected at 0, 30, 60, 90, 120, 150, and 180 minutes, respectively. The supernatant was discarded following centrifugation at 3000 rpm for 5 minutes, and the wet weight (Wt) was measured after the residual liquid on the surface was eliminated using filter paper. The sample's starting weight (W) was determined using a precision balance, while the weight of the EP tube (W0) was calibrated independently. The swelling ratio was determined using the formula SR (%) = [(Wt-W0)/W]×100%, with n≥3 parallel samples established in each group.

In vitro enzyme degradation experiment: The pre-weighed (W0) freeze-dried microgels were immersed in PBS solution containing 1% (w/v) lysozyme/hyaluronidase and a non-enzyme control group, respectively, and agitated at 37°C (80 rpm). The degrading medium was refreshed every 48 hours. Samples were collected weekly, rinsed thrice with deionized water, and subjected to freeze-drying. The deterioration rate was determined using the formula (Wt/W0)×100%, where Wt represents the net weight of the freeze-dried sample post-degradation. The experiment was conducted thrice.

**Biocompatibility of Cel/Lipo+/BHMs microgel**

To assess the biocompatibility of the injectable Cel/Lipo+/BHMs microgels, we co-cultured the microgels with C28/I2 cells, subsequently examining chondrocyte viability using live-dead cell labeling and evaluating the microgels' cytotoxicity to chondrocytes via the CCK-8 assay. Particularly as mentioned below.

Live/Dead cell staining: C28/I2 cells were plated at a density of 5×10^4^ cells per dish in a confocal culture dish. Upon achieving 70-80% cell fusion, Cel/Lipo+, BHMs, and Cel/Lipo+/BHMs microgels were subsequently introduced, with untreated cells serving as the control group. Following co-culture at 37°C for 24 to 48 hours, the media was removed and the cells were washed three times with PBS (pH 7.4). The detection solution comprising 2μM Calcein AM and 4μM propidium iodide was administered and incubated in the dark for 30 minutes. Fluorescence microscopy (Zeiss) was employed to examine the staining at 488 nm/530 nm (green) and 561 nm/617 nm (red) channels, respectively. Five fields of view were randomly chosen for imaging analysis.

CCK-8 experiment: C28/I2 cells were seeded into 96-well plates at a density of 5×10³. Upon achieving 70-80% cell fusion, BHMs, and Cel/Lipo+/BHMs microgels were subsequently introduced. C28/I2 cells that were typically grown served as the control group. Following co-culture for 24, 48, and 72 hours, the medium in each dish was removed and rinsed three times with PBS. Ten microliters of CCK-8 solution and ninety microliters of blank medium were added to each well and incubated in the dark at 37°C for one hour. The optical density of each well was assessed at 450 nm utilizing a multifunctional microplate reader.

**Evaluation of blood compatibility**

Various materials (Cel/Lipo+, BHMs, Cel/Lipo+/BHMs) were pre-incubated with normal saline (0.9% NaCl) at 37°C for 48 hours to generate the material extract. The whole blood of New Zealand rabbits (obtained from the ear vein) was centrifuged at 2000× g for 10 minutes to isolate red blood cells (RBC). Following three washes with normal saline (centrifugation conditions: 1500×g for 5 minutes), a 5% RBC suspension was produced. Experimental groups: negative control (RBC + 0.9% NaCl), positive control (RBC + 0.1% Triton X-100), Cel/Lipo+ group (100 μL extract + RBC + 0.9% NaCl), BHMs group (100 μL extract + RBC + 0.9% NaCl), Cel/Lipo+/BHMs group (100 μL extract + RBC + 0.9% NaCl). Following incubation at 37°C for 1 hour, the supernatant was centrifuged at 1500×g for 5 minutes, and 200 μL of the supernatant was transferred to a 96-well plate (n = 3). The absorbance at 540 nm (A) was quantified using a microplate reader.

Hemolysis rate =[(A sample-A negative)/(A positive-A negative)]×100%

In which A sample, A negative, and A positive were the absorbance values of the sample, negative, and positive control groups, respectively.

**Analysis of proliferation and migration of C28/I2 cells**

Fluorescence labeling of the chondrocyte cytoskeleton was employed to assess cell growth. C28/I2 cells were inoculated in a confocal dish at a density of 5×10^4^. Upon achieving 70-80% cell fusion, Cel/Lipo+, BHMs, and Cel/Lipo+/BHMs microgels were subsequently introduced. Cultured C28/I2 cells served as the control group. Following co-culture for 24 and 48 hours, the culture medium in each dish was removed, washed thrice with PBS, and fixed with 4% paraformaldehyde for 20 minutes. Following fixation, PBS was rinsed three times, and 0.1% Triton-X-100 was introduced to each dish for 5 minutes. After washing the PSB thrice, 2% BSA was introduced for a 30-minute blocking period, followed by the addition of 1 μL phalloidin working solution to each dish, which was incubated in the dark for 75 minutes. The cells were rinsed thrice with PBS, re-stained with DAPI, and incubated at ambient temperature for 3 minutes in the dark. Following three washes with PBS, the cells were examined and photographed using a fluorescence microscope, and the cellular proliferation activity was assessed based on the morphological properties of the cytoskeleton.

The impact of Cel/Lipo+/BHMs drug-loaded microgels on cellular migration was assessed using a scratch healing test. C28/I2 cells (3×10^5^ cells per well) were inoculated in 6-well plates and grown in DMEM media supplemented with 10% fetal bovine serum for 24 hours to achieve monolayer confluence. A 200 μL sterile pipette tip, oriented perpendicular to the long axis of the orifice plate, was employed to artificially generate linear scratches. After the gentle washing with PBS to eliminate the exfoliated cells, the medium containing Cel/Lipo+, BHMs, and Cel/Lipo+/BHMs was added sequentially. The cells were grown at 37°C and 5% CO2 for 0, 12, and 24 hours. The control group was grown with an equivalent volume of complete medium. Scratch images were acquired using an inverted optical microscope at each time point, and the alteration in the scratch area was quantitatively assessed using ImageJ software.

Wound closure rate (%) = [(WA_0_-WA_t_)/WA_0_]×100%

WA_0_ is the initial scratch area, and WA_t_ is the residual scratch area at time t.

**The effect of Cel/Lipo+/BHMs microgels on intracellular reactive oxygen species**

To ascertain if the lubricating microgel can diminish intracellular ROS, C28/I2 cells were plated in a confocal dish at a density of 5×10^4^ cells/dish and incubated overnight to facilitate cell adhesion. The cells were stimulated with LPS (1μg/mL) to generate excessive ROS, whereas the experimental group received a pre-prepared drug medium containing Cel/Lipo+, BHMs, and Cel/Lipo+/BHMs microgels for a duration of 6-12 hours. The intracellular ROS levels were assessed using fluorescent labeling with the DCFH-DA kit. DCFH-DA is a permeable probe for the detection of intracellular reactive oxygen species (Excitation/Emission = 488/525 nm). The cells were treated with DCFH-DA (10 μM) in darkness for 30 minutes, followed by three washes with PBS. Following three instances of PBS cleaning, the fluorescence distribution within the cells was examined using a fluorescence microscope and documented by photography. The fluorescence intensity was quantified with Image J. Simultaneously, flow cytometry was employed to objectively assess intracellular ROS clearance and determine the ROS clearance rate. All studies were conducted using three biological replicates.

**Macrophage polarization mediates the regulation of inflammatory microenvironment**

To investigate the impact of microgels on macrophage polarization, RAW264.7 cells were cultured in a 12-well plate at a density of 2×10^5^cells per well. After the adhesion of the cells to the wall, the drug medium, including Cel/Lipo+, BHMs, and Cel/Lipo+/BHMs, was administered for 12 hours. The cells were subsequently exposed to 100 ng/ml LPS for 24 hours. The group treated with 100 ng/ml LPS was designated as the positive control (M1). Flow cytometry was employed to assess the impact of various treatments on macrophage polarization. The macrophages, both pre-and post-treatment, along with the control macrophages, were harvested and treated with 100 μl PBS (containing F4/80/FITC, CD86/APC antibody, and CD206/PE antibody) at 4°C for 30 minutes and at room temperature for 1 hour, respectively. The expression levels of CD86 and CD206 were assessed by flow cytometry.

Immunofluorescence staining was employed to further assess the impact of microgels on macrophage polarization. Initially, RAW264.7 cells were incubated overnight at a density of 5×10^4^ cells per dish and subsequently stimulated with complete media containing LPS at a concentration of 1 μg/mL. The control group consisted of cells that were treated normally. Simultaneously, Cel/Lipo+, BHMs, and Cel/Lipo+/BHMs microgels were subjected to a 24-hour treatment. The cell samples were fixed in 4% paraformaldehyde for 30 minutes, permeabilized with 0.1% Triton X-100 for 5 minutes, and subsequently blocked with 2% BSA for 30 minutes. Following blocking, the samples were incubated with primary antibodies (F4/80, CD86, or CD206, diluted at 1:200) at 4°C overnight. The following day, PBS was rinsed three times and thereafter incubated with the appropriate fluorescently tagged secondary antibody at room temperature for 60 minutes. Following PBS washing, phalloidin was introduced for cytoskeletal staining. The nucleus was stained with DAPI for 15 minutes, thereafter rinsed with PBS, mounted, and examined under a fluorescence microscope for picture collection. CD86-positive cells (green fluorescence) represented M1 macrophages, while CD206-positive cells (red fluorescence) indicated M2 macrophages.

To assess the impact of modulating the immunological microenvironment on the expression of inflammatory factors, TNF-α and IL-1β were examined using immunofluorescence labeling. RAW264.7 cells were seeded in 12-well plates at a density of 2×10^5^ per well. After the adhesion of the cells to the wall, 1μg/ml LPS and various pharmacological agents were administered to induce the production of inflammatory factors and facilitate intervention. Conventional RAW264.7 cells were utilized as the control group. Following 24 hours of co-culture, the expression of inflammatory factors in each group was assessed using immunofluorescence staining. The precise steps are as follows: The culture medium from each group was discarded, followed by washing with PBS, fixation using 4% paraformaldehyde, and permeabilization with 0.1% Triton X-100 for 5 minutes. Subsequently, blocking was performed with BSA, and TNF-α and IL-1β primary antibodies (dilution ratio of 1:200) were added and incubated overnight at 4°C. After washing with the primary antibody, incubation with a fluorescent secondary antibody was conducted for 1 hour. Following PBS cleansing, phalloidin was administered for cytoskeletal staining, and after incubation, DAPI was introduced for nuclear staining for 3 minutes. After PBS cleaning, the sections were mounted, and the fluorescence expression in each group was ultimately examined using a fluorescence microscope.

**Transcriptomics and validation of macrophage reprogramming**

To examine the transcriptional reprogramming of macrophages, RAW264. Seven cells were seeded in 10 cm dishes at a density of 5×10⁵ cells per dish and stimulated with 500 ng/mL LPS for 24 hours to create an inflammatory model, comprising five treatment groups (Control, Model, Cel/Lipo+, BHMs, and Cel/Lipo+/BHMs) alongside an unstimulated PBS control group. Subsequently, total RNA was extracted using TRIzol reagent and quantified for purity using NanoDrop spectrophotometry. For transcriptome sequencing, 1 μg of DNase I-digested RNA per sample was utilized to construct strand-specific libraries using the NEBNext Ultra II Kit, which involved mRNA enrichment, ultrasonic fragmentation, double-stranded cDNA synthesis, adapter ligation, and PCR amplification, culminating in paired-end 150 bp sequencing on the Illumina NovaSeq 6000 platform. Raw sequencing data were subjected to quality control using Trimmomatic, aligned to the GRCm39 genome with HISAT2, and differentially expressed genes were identified through DESeq2, emphasizing bioinformatic analyses on NF-κB/STAT pathway genes and polarization markers, including KEGG/GO enrichment and pathway activity scoring via gene set enrichment analysis (GSEA). To validate the findings, extracted RNA underwent reverse transcription to cDNA for quantitative PCR analysis of Nos2, IL10, TNFα, and reference rRNA expression. Relative expression levels were determined using the 2−ΔΔCt method to assess the synergistic impact of Cel/Lipo+/BHMs on the upregulation of M2-polarization-associated reparative genes and the downregulation of M1-polarization-associated inflammatory genes compared to control groups.

**The effect of Cel/Lipo+/BHMs microgel on the expression of C28/I2 function-related proteins induced by IL-1β**

The impact of the microgel on proteins associated with cartilage metabolism was confirmed using immunofluorescence labeling. The particulars are as follows: C28/I2 cells were inoculated into a confocal dish at a density of 5×10^4^ cells per well. Following cell adherence, a 10 ng/ml IL-1β solution was introduced to produce the osteoarthritis cartilage model. Following a 24-hour induction period, DMEM complete medium (Control group), Cel/lipo+, BHMs, and Cel/lipo+/BHMs microspheres were included. The standard cultivated C28/I2 cells served as the control group (NonIL-1β group). Following 48 hours of co-culture, the expression of proteins associated with cartilage metabolism in each group was assessed by immunofluorescence labeling. The precise procedure involved: discarding the culture medium from each dish, rinsing with PBS, and adding 1 mL of 4% paraformaldehyde to each dish for 25 minutes at ambient temperature. Upon completion of fixation, PBS was rinsed three times, followed by the addition of 1 mL of 0.1% Triton X-100 to each dish for 5 minutes at ambient temperature. Following three washes with PBS, 2% BSA was introduced for blocking for 30 minutes, after which the primary antibodies for Col2 and MMP-13 (dilution ratio of 1:200) were added for overnight incubation at 4°C. The next day, the primary antibody was removed, followed by three washes with PBS, after which the appropriate fluorescent secondary antibody (dilution ratio of 1:300) was applied and incubated at room temperature for 2 hours. The second antibody was eliminated, PBS was rinsed three times, and DAPI was introduced for a 5-minute incubation at room temperature and in the absence of light. Following three washes with PBS, fluorescence expression in each group was examined using a fluorescence microscope.

To investigate the direct effects of reprogrammed inflammatory macrophages on inflammation suppression and cartilage repair, chondrocytes were seeded in 6-well plates at 5×10⁴ cells/well. After 24 h of adherence, cells were stimulated for 24 h with 10 ng/mL IL-1β to establish an inflammatory model. RAW264.7 macrophages were divided into five treatment groups: Control, Model, Cel/Lipo+, BHMs, and Cel/Lipo+/BHMs. Conditioned media (CM) from each macrophage group were collected, centrifuged at 12,000g for 10 min, and filter-sterilized through 0.22 μm membranes. The medium of inflamed chondrocytes was then replaced with a mixture containing 30% (v/v) macrophage CM. Following co-culture, immunofluorescence was performed to evaluate Col2 and MMP13 protein expression. Total RNA was extracted from chondrocytes for qPCR analysis of Col2 and MMP13 mRNA expression. Conditioned medium from M1 macrophages induced by 100 ng/mL LPS served as a positive control. All experiments were independently repeated three times.

For macrophage migration assessment towards chondrocyte injury sites, a Transwell system was employed. C28/I2 chondrocytes were seeded in the lower chambers of 24-well plates and pre-stimulated with 10 ng/mL IL-1β for 48 h to simulate an injury microenvironment. RAW264.7 cells from the five treatment groups were resuspended in serum-free DMEM at 2×10⁵ cells/mL. A 200 μL aliquot of each cell suspension was added to the upper chambers (8 μm polycarbonate membrane). The lower chambers received either: 1) Control group: CM from IL-1β-pre-stimulated chondrocytes; or 2) Experimental groups: chondrocyte CM supplemented with corresponding pretreatment agents (final concentration: 50 μg/mL for Cel/Lipo+, 200 μg/mL for BHMs or Cel/Lipo+/BHMs). After 24 h of migration, non-migrated cells were removed from the upper membrane surface. Migrated cells on the membrane underside were fixed with 4% paraformaldehyde, stained with crystal violet, and quantified by counting cells in six randomly selected fields per membrane.

**Biodistribution and articular cavity retention of Cel/Lipo+/BHMs in vivo**

Seven days post the successful implementation of the OA model, the rats in the experimental group were administered a single injection (50μL, 1mg/mL) of Cel/Lipo+ and Cel/Lipo+/BHMs microgel suspension containing DiR near-infrared dye into the knee joint cavity. Timing imaging was conducted with a small animal in vivo imaging system (IVIS Spectrum, PerkinElmer, excitation/emission wavelength 745/800 nm, exposure duration 500 ms) before surgery (baseline) and from 0 to 28 days post-surgery. The fluorescence intensity of the joint region was measured using ROI analysis software (Living Image 4.5) to confirm the microgel's long-term retention properties within the joint cavity.

**Construction and treatment of the rat OA model**

SD rats were sedated using an isoflurane gas anesthesia equipment, with the level of anesthesia assessed through continuous monitoring of respiration rate and toe reflex. A unilateral osteoarthritis model was created with the accurate injection of sodium iodoacetate (MIA, Sigma, 2 mg/50 μL normal saline) into the right knee joint cavity. Immediately following injection, passive flexion and extension of the joints were executed 15 times (amplitude > 90°, frequency 30 times/min) to facilitate drug diffusion. Following 7 days of modeling, the subjects were randomly allocated into 5 groups (n=5/group): sham operation group (needle puncture without injection), OA model group (normal saline 50 μL), Cel/Lipo+ group (50 μg/mL, 50 μL), BHMs group, and Cel/Lipo+/BHMs composite microgel group. Each group received intra-articular injections weekly for five weeks.

**Radiographic evaluation**

Following a 5-week treatment period, the knee joints of rats were harvested and preserved in a 4% (V/V) paraformaldehyde solution. The acquired knee joint specimens were examined with Quantum GX Micro-CT (PerkinElmer, MA, USA). The joint spacing width was assessed in both anterior and posterior orientations, and the knee joint was obtained for in vitro Micro CT analysis. Films in the anteroposterior (AP) and lateral (LAT) orientations were chosen for scanning. The micro-CT scanning parameters were as follows: Voltage: 90 kV; Current: 88 mA; Pixel size: 90.0 mm. Representative three-dimensional reconstructed pictures were acquired and processed with Mimics software.

**Histopathological analysis**

Joint swelling was measured with a vernier caliper on either side adjacent to the tibial plate. Knee widths were recorded at days 0, 7, 14, 21,28, and 35 to reflect the degree of knee swelling. The value is expressed in millimeters (mm), and the calculation formula for the change of knee width (ΔW) was as follows: ΔW = (dT-d0)/d0 × 100%

For the histological study, following 5 weeks of administration, the acquired joint specimens were fixed in 4% paraformaldehyde and decalcified using a 10% EDTA decalcification solution. Paraffin-embedded and sectioned to a thickness of 5 μm. Histological properties of the tissue were analyzed using H&E staining, safranine fast green staining, and toluidine blue staining to assess the microgel's efficacy in osteoarthritis remission and cartilage regeneration. Paraffin sections were evaluated and quantitatively examined following the Mankin standard.

**Immunohistochemical evaluation**

Paraffin sections were baked overnight at 60°C, followed by a 10-minute immersion in a xylene I, II gradient for dewaxing, a 5-minute rehydration in anhydrous ethanol to a 75% gradient ethanol, and a 3-minute soaking in PBS to finalize the procedure. The epitope was revealed by a high-pressure thermal repair technique. Following natural cooling to ambient temperature, the endogenous peroxidase was inhibited by incubation with a 3% H_2_O_2_ methanol solution at room temperature for 20 minutes. The cells were incubated at 7°C for 30 minutes in a 5% BSA blocking solution. Subsequent to the application of the blocking solution, rabbit anti-(MMP13, TNF-α, IL-1β, Col2, and Aggrecan) monoclonal primary antibody (1:200, Abcam ab9535) was incubated overnight at 4°C. After rewarming the following day, the cells were subjected to three washes with TBST (5 minutes each) and then incubated with HRP-conjugated goat anti-rabbit secondary antibody at 37°C for one hour. Following TBST washing, the DAB color reagent was applied and protected from light for 3-5 minutes to regulate color intensity. Hematoxylin was re-stained for thirty seconds. Following differentiation with 1% hydrochloric acid in ethanol, the water reverted to blue. Gradient ethanol dehydration, xylene clearing, and neutral gum sealing were performed following brilliant field microscopy observation, with quantitative analysis of positive regions conducted using ImageJ software.

**Detection of biosafety and immunogenicity in vivo**

One month after injection, blood was collected from the tail vein, and serum was separated to detect liver and kidney function indicators-alanine aminotransferase (ALT), aspartate aminotransferase (AST), alkaline phosphatase (ALP) to evaluate hepatotoxicity, Creatinine (CREA), UREA, uric acid (UA) to evaluate nephrotoxicity; the animals were sacrificed at the end of 1 month. The heart, liver, spleen, lung, kidney, and knee joint of the injection side were fixed in 4% paraformaldehyde, paraffin-embedded, and sectioned for H&E staining (histopathological damage was observed). The knee joint tissues were collected for immunohistochemical staining to evaluate macrophage infiltration (CD68), neutrophil infiltration (MPO), and complement deposition (Cd3).

**Statistical analysis**

The experimental data were presented as the mean±standard deviation (SD). Statistical analysis was performed using one-way ANOVA followed by Tukey’s post-hoc test to assess the significance levels of the software Origin 2022. Statistical significance was defined as **p* < 0.05, ***p* < 0.01, ****p* < 0.001.

**Supplementary Figures**

**
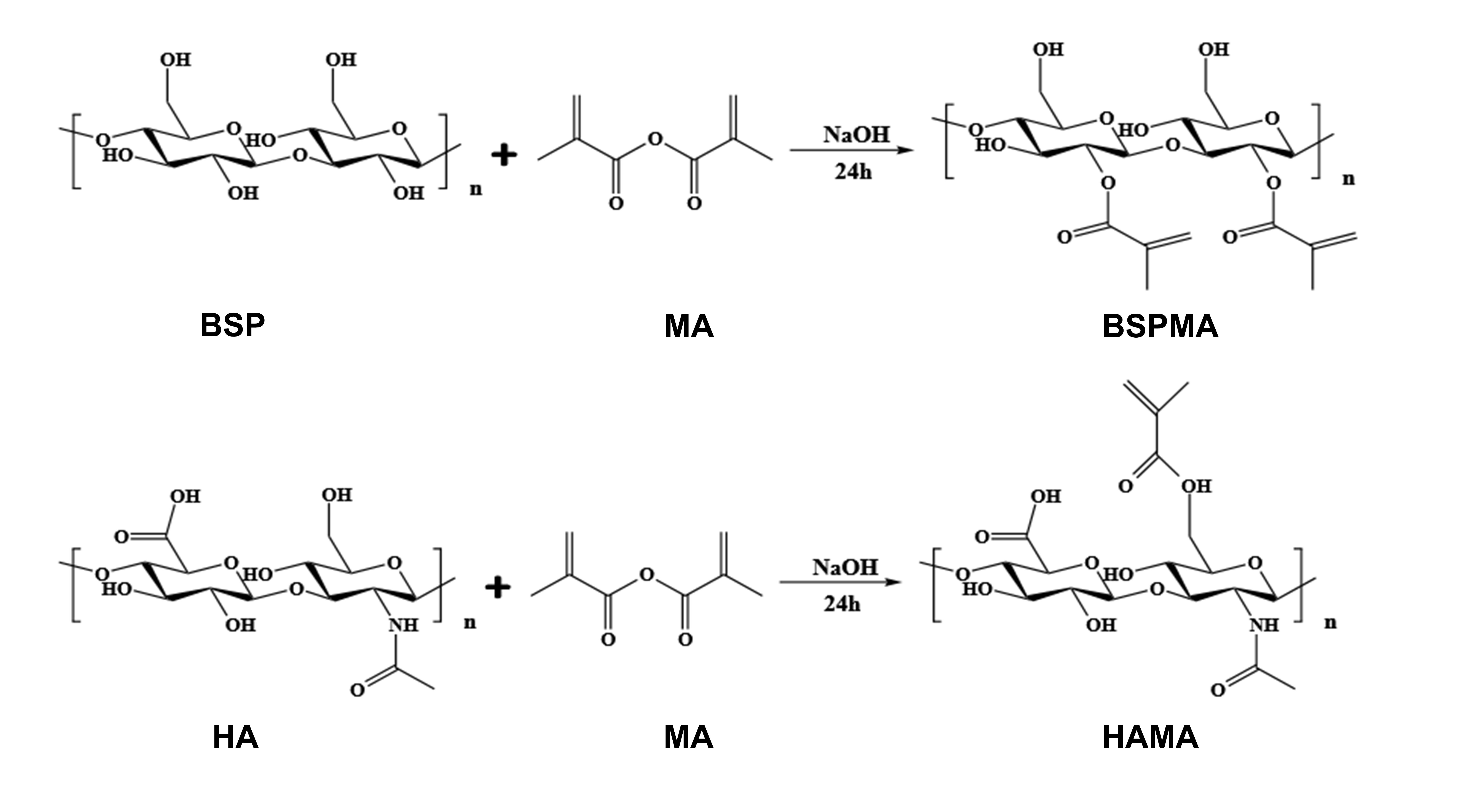
Figure S1.** The synthetic routes of BSPMA and HAMA

**
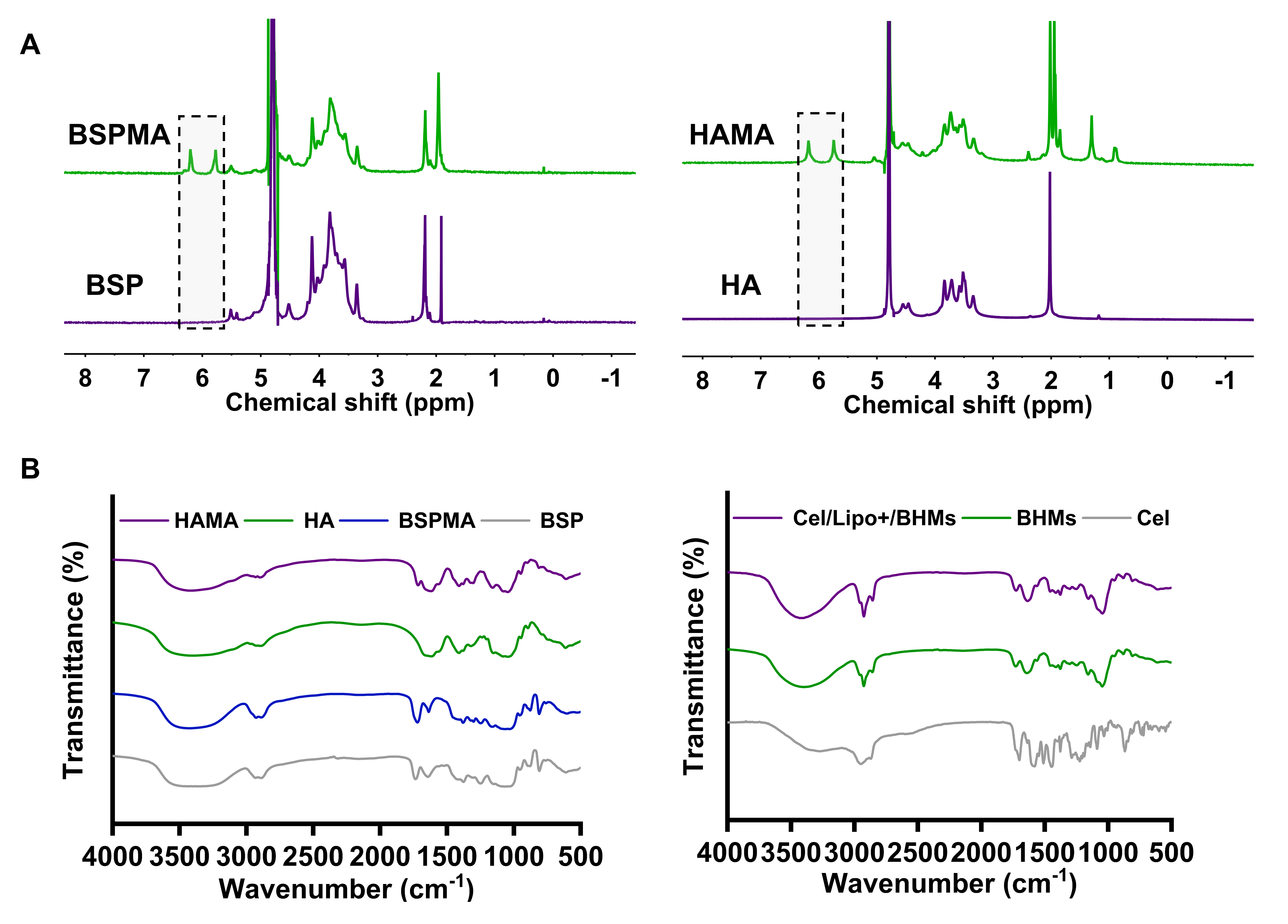
**

**Figure S2.** Characterization of BSPMA and HAMA. (A) The ^1^H-NMR spectrum of BSP and BSPMA, HA and HAMA. Peaks corresponding to methacrylate are noted by black dotted rectangles. (B) FTIR images of BSP, BSPMA, HA, and HAMA. FTIR diagram of Cel, BHMs, and Cel/Lipo+/BHMs.

**
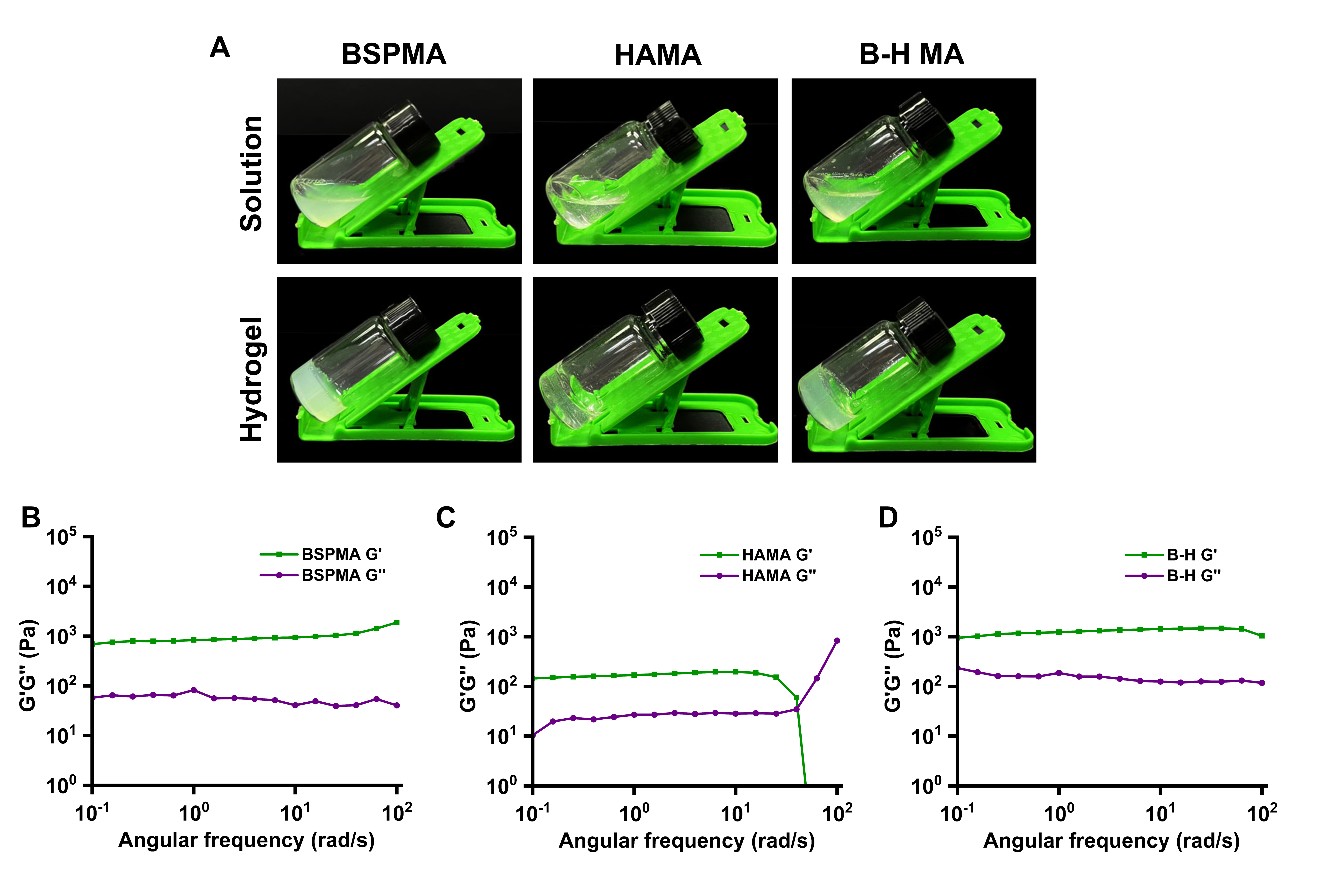
Figure S3.** (A) The vial inversion test results for BSPMA, HAMA, and B-H MA. (B, C, D) The rheological characterization results for BSPMA, HAMA, and B-H MA.

**
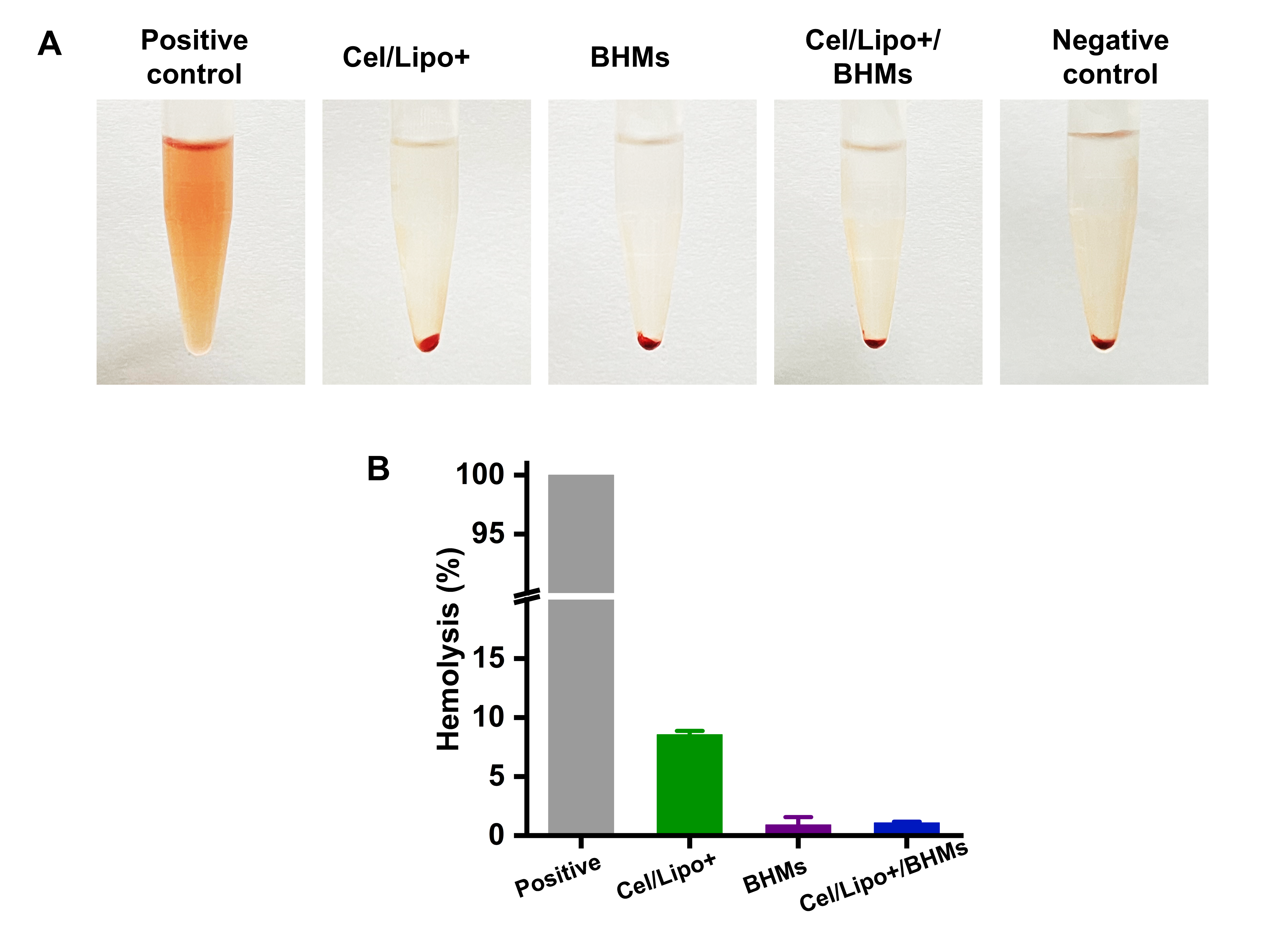
**

**Figure S4.** (A) Representative hemolysis assay images. (B) Hemolysis rate.


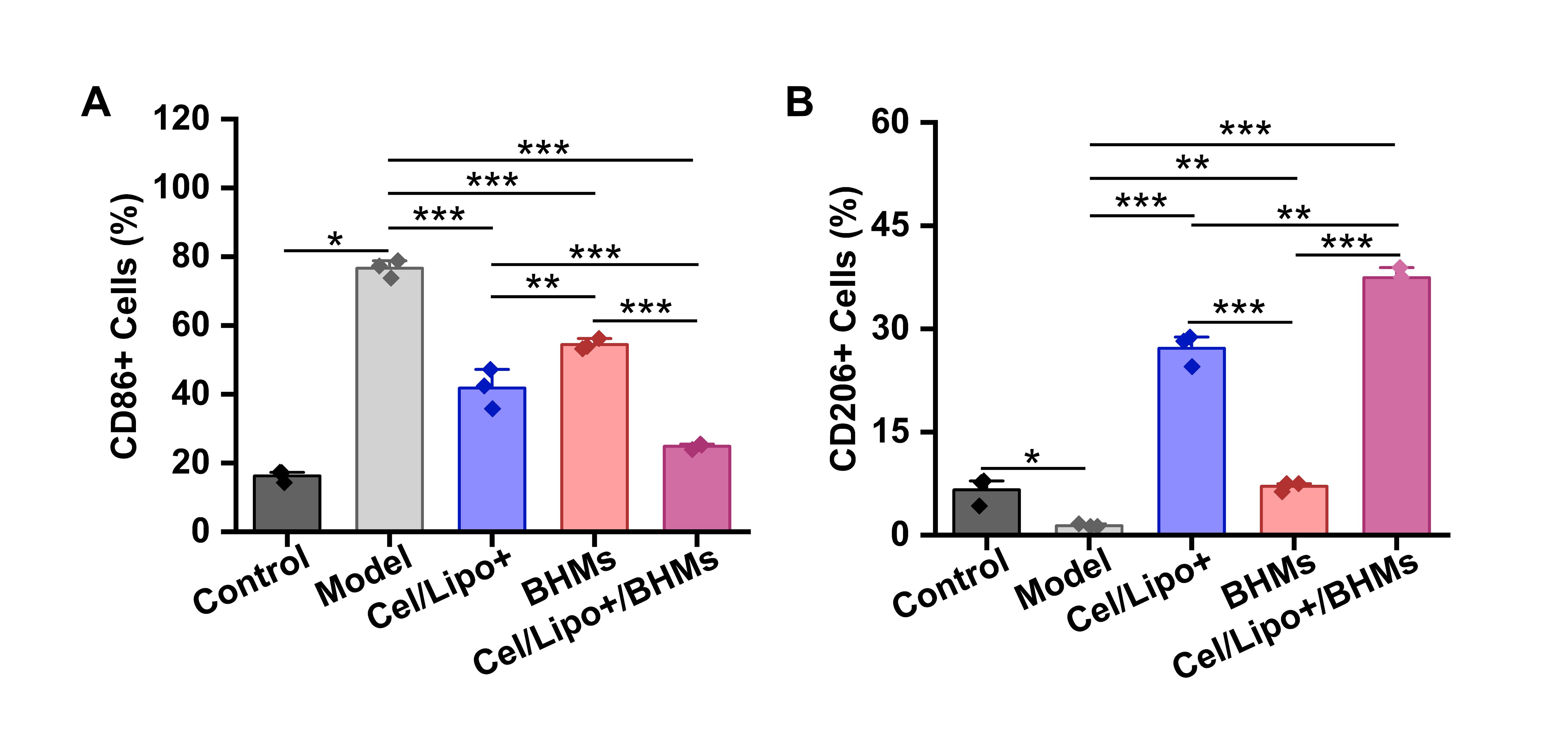
**Figure S5.** Results of flow cytometric quantification of macrophage polarization. (A) CD86-positive expression. (B) CD206-positive expression. Data represent mean ± SD. **p*< 0.05, ***p*< 0.01, ****p*<0.001.

**
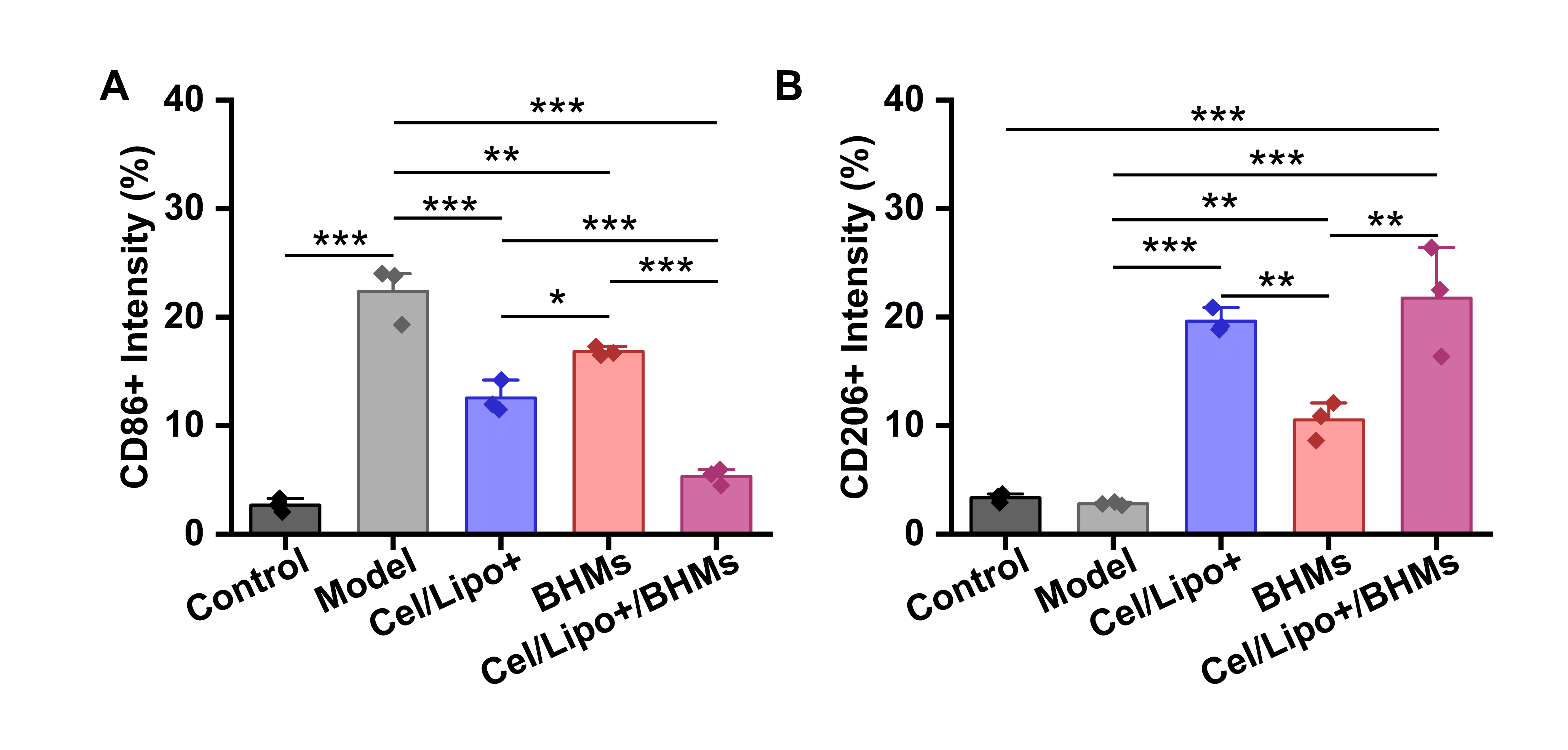
**

**Figure S6.** Quantification of immunofluorescence expression for CD86 and CD206**. (**A) M1 macrophage expression diagram (n=3). (B) M2 macrophage expression diagram (n =3). Data represent mean ± SD. **p*< 0.05, ***p*< 0.01, ****p*<0.001.


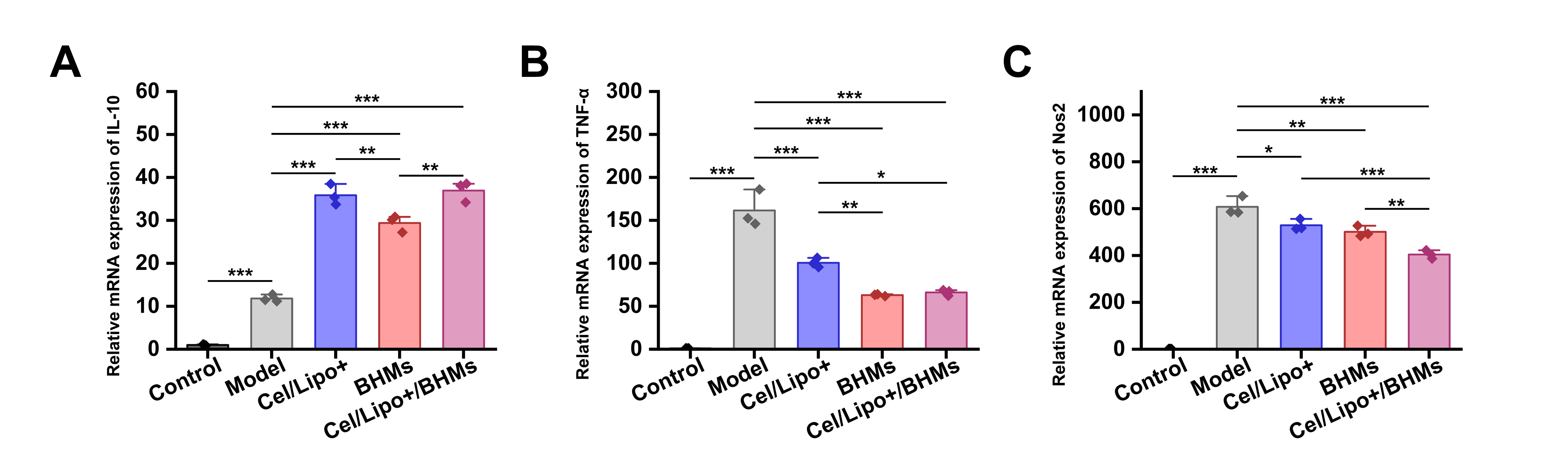
**Figure S7.** The expression levels of macrophage polarization-related indicators (A- IL-10, B-TNF-α, C-Nos2 ) genes after treatment in different groups.


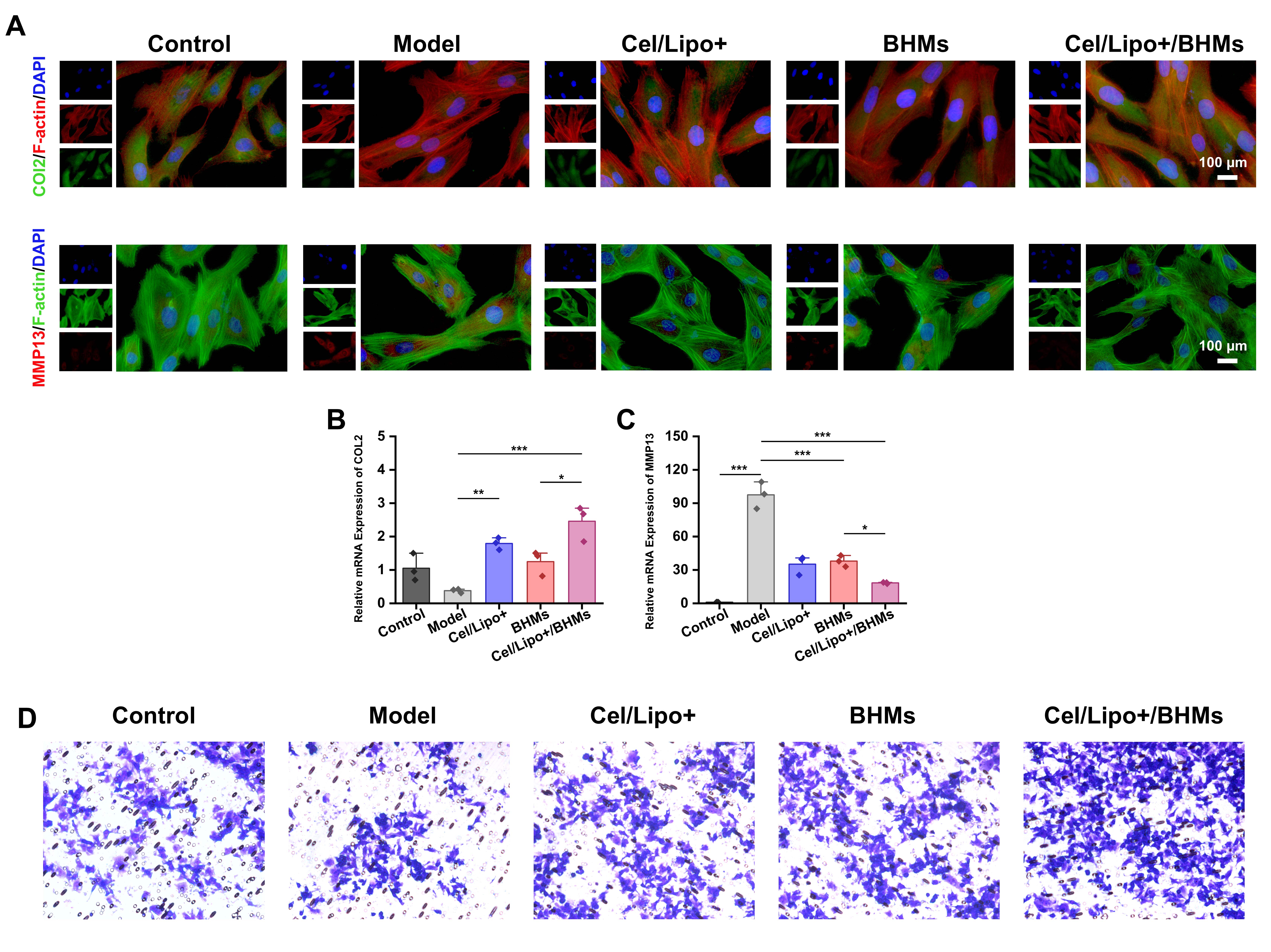


**Figure S8.** (A) Immunofluorescence analysis of COL2 and MMP13 genes in chondrocytes in the macrophage-chondrocytes co-culture system. Scale bar:100 μm. (B, C) The expression levels of COL2 and MMP13 genes in chondrocytes were observed after stimulation with RAW264.7 cell culture supernatant. (D) The Transwell double-layer model was used to simulate the migration of macrophages to cells in the injured area during cartilage injury. n≥3, Data represent mean±SD. *p< 0.05, **p< 0.01, ***p<0.001.


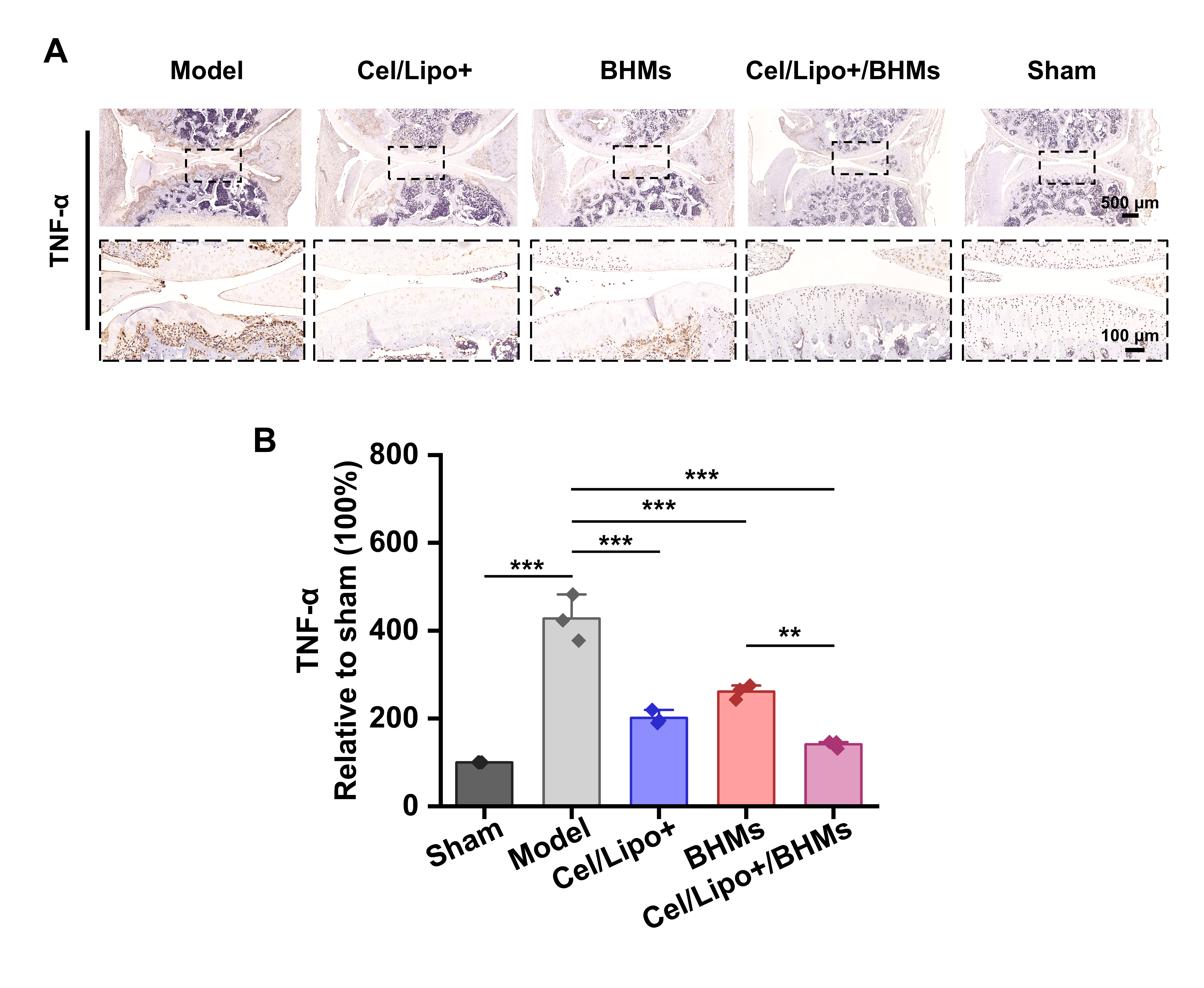
**Figure S9.** (A) Representative immunohistochemical images of TNF-α.(B) Quantitative analysis of TNF-α positive expression. Scale bar:500,100 μm. (n=3). Data represent mean±SD. **p*< 0.05, ***p*< 0.01, ****p*<0.001.


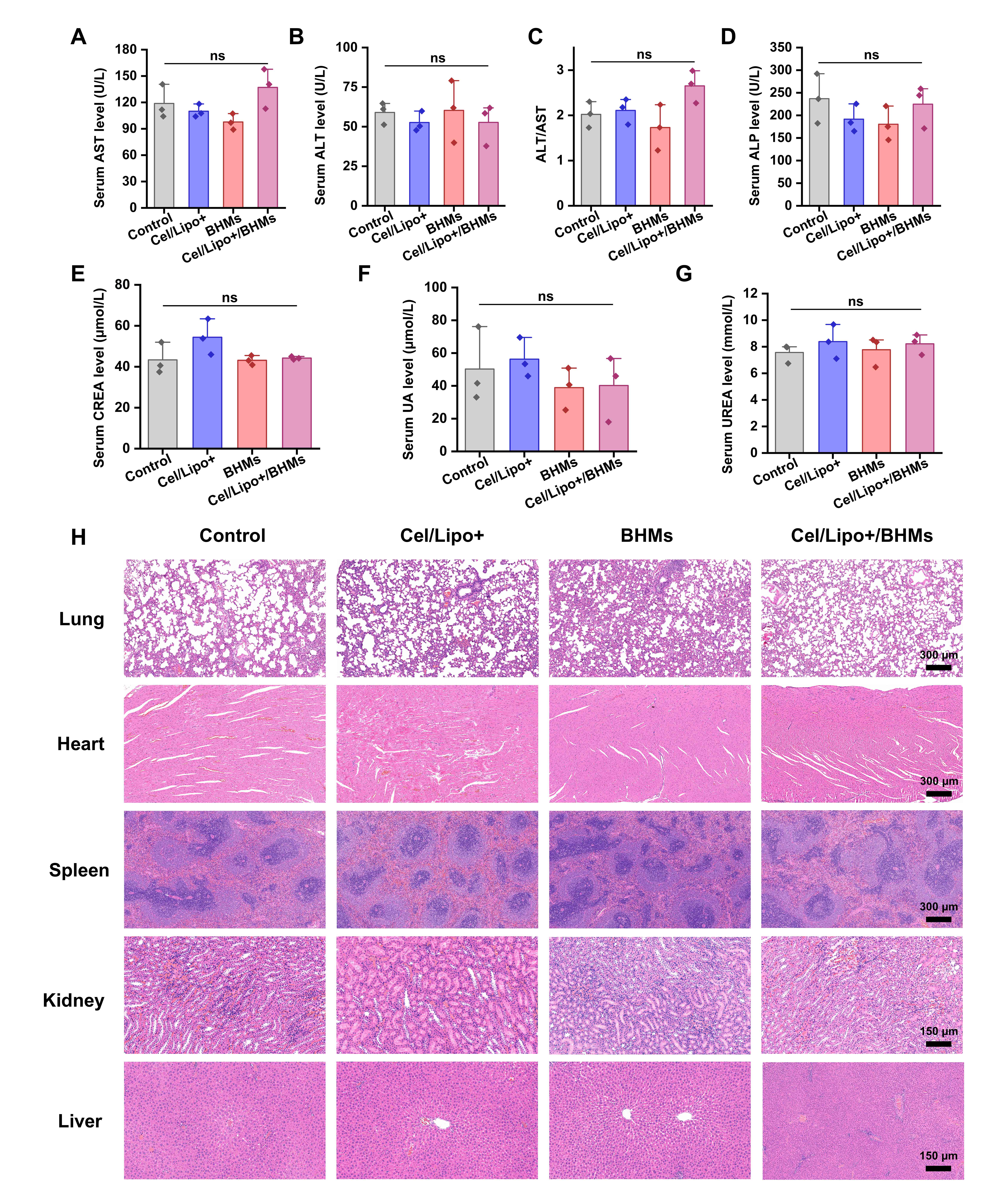


**Figure S10.** In vivo safety evaluation. Biochemical indicators of liver and kidney functions: (A) AST, (B) ALT, (C) ALT/AST, and (D) ALP. (n = 3). (E) CREA, (F) UA, (G) UREA. (n = 3). (I) H&E-stained sections of major organs (Scale bar: 150 µm、300 µm). Data represent mean±SD. Ns means no significant difference.

**
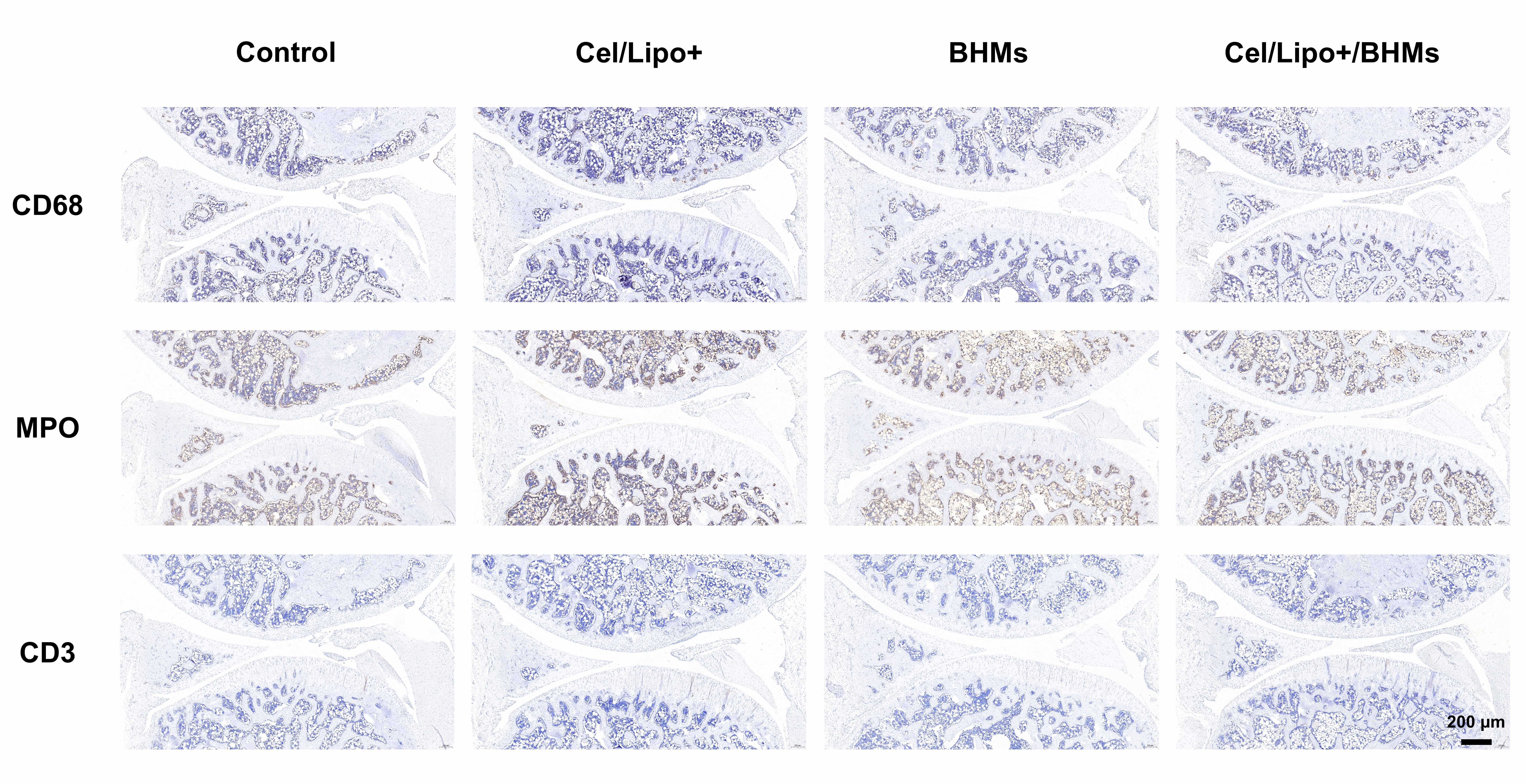
**

**Figure S11.** Analysis of CD68, MPO, and CD3 immunohistochemical staining. (Scale bar:200 μm.)

**Table S1: Primers used in the study.**

| **Transcription** | **Primer sequences** | |
| --- | --- | --- |
| Nos2 | Forward primer | GTTCTCAGCCCAACAATACAAGA |
|  | Reverse primer | GTGGACGGGTCGATGTCAC |
| IL-10 | Forward primer | GCTCTTACTGACTGGCATGAG |
|  | Reverse primer | CGCAGCTCTAGGAGCATGTG |
| TNF-α | Forward primer | CAGGCGGTGCCTATGTCTC |
|  | Reverse primer | CGATCACCCCGAAGTTCAGTAG |
| Col2 | Forward primer | GCCAGGATGCCCGAAAATTA |
|  | Reverse primer | CGTCATACCCTCCAGCCATC |
| MMP13 | Forward primer | GACAAGCAGTTCCAAAGGCTAC |
|  | Reverse primer | GGATGCTTAGGGTTGGGGTC |
| GAPDH | Forward primer | CATCACTGCCACCCAGAAGACTG |
|  | Reverse primer | ATGCCAGTGAGCTTCCCGTTCAG |
